# Characterizing the salivary RNA landscape to identify potential diagnostic, prognostic, and follow‐up biomarkers for breast cancer

**DOI:** 10.1002/1878-0261.70101

**Published:** 2025-09-01

**Authors:** Nicholas Rajan, Irina Primac, Emre Etlioglu, Laurens Debruyne, Ann Janssen, Magy Sallam, Kevin Tabury, Roel Quintens, Wiebren Tjalma, Mohammed Abderrafi Benotmane

**Affiliations:** ^1^ Radiobiology Unit, Nuclear Medical Applications Institute, Belgian Nuclear Research Centre SCK CEN Mol Belgium; ^2^ Multidisciplinary Breast Clinic, Gynaecological Oncology Unit, Department of Obstetrics and Gynaecology Antwerp University Hospital Edegem Belgium

**Keywords:** biomarkers, breast cancer, lncRNA, liquid biopsy, molecular diagnostics and prognostics, salivary biomarkers

## Abstract

Breast cancer (BC) diagnostics and prognostics traditionally rely on invasive tissue biopsies, presenting limitations for large‐scale screening and continuous patient monitoring. Salivary biomarkers have recently emerged as a compelling noninvasive and accessible alternative, offering significant potential for population‐level screening and long‐term monitoring of BC. In this study, we conducted a comprehensive salivary transcriptomic profiling of BC patients using high‐throughput RNA sequencing. Our analysis captured a wide spectrum of RNA species, including mRNAs, lncRNAs, miRNAs, and snRNAs, highlighting their collective contributions in the molecular landscape of BC patient saliva. We identified robust human gene expression signatures that distinguish BC patients from healthy individuals. Importantly, we discovered RNA profiles that were differentially expressed relative to control samples, enabling the discrimination of noninvasive, invasive, and mixed histological types, as well as hormone receptor‐positive molecular subtypes. These salivary markers showed substantial concordance with established tumor gene expression datasets, strengthening their potential relevance in clinical stratification. Furthermore, we identified subsets of salivary genes associated with nodal involvement and others linked to poor survival outcomes, highlighting their potential as prognostic indicators. A prospective follow‐up analysis revealed a decline in the expression of several cancer‐related salivary transcripts 1‐year posttreatment, indicating that salivary RNA might also reflect treatment response over time. This study establishes a proof‐of‐concept for salivary RNA biomarkers as a versatile, accessible, and robust tool for BC diagnosis, prognosis, and follow‐up, paving the way for innovative biomarker‐driven strategies in oncology.

## Graphical abstract

This study explores salivary RNA for breast cancer (BC) diagnosis, prognosis, and follow‐up. High‐throughput RNA sequencing identified distinct salivary RNA signatures, including novel transcripts, that differentiate BC from healthy controls, characterize histological and molecular subtypes, and indicate lymph node involvement. Key transcripts also show prognostic value and dynamic changes posttreatment, emphasizing salivary RNA as a promising noninvasive biomarker tool.

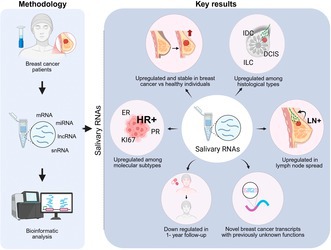

**Abbreviations**
ASCO: american society of clinical oncology
BC: breast cancer
CI: confidence interval
Ctrl(s): controls (used for healthy)
CV: coefficient of variation
DCIS: ductal carcinoma *in situ*
DE: differentially expressed
DEA: differential expression analysis
DEGs: differentially expressed genes
ELISA: enzyme‐linked immunosorbent assay
EMT: epithelial‐mesenchymal transition
ENSG IDs: ensembl gene identifiers
ER: estrogen receptor
FC: fold change
FC_M.T: fold change metastatic vs. tumor
FC_T.C: fold change tumor vs. control
FDR: false discovery rate
FFPE: formalin‐fixed, paraffin‐embedded
FISH: fluorescence *in situ* hybridization
FL: follow‐up
HER2: human epidermal growth factor receptor 2
HOMD: human oral microbiome database
HR: hormone receptor
IC: invasive carcinoma not otherwise specified
IDC: invasive ductal carcinoma
IHC: immunohistochemistry
ILC: invasive lobular carcinoma
lncRNAs: long noncoding RNAs
log_2_FC: log_2_ fold change
Lum‐A: luminal A
Lum‐B: luminal B
miRNAs: microRNAs
mt‐RNAs: mitochondrial RNAs
NCCN: national comprehensive cancer network
ncRNA: noncoding RNA
NGS: next‐generation sequencing
OS: overall survival
PR: progesterone receptor
qRT‐PCR: quantitative reverse transcription polymerase chain reaction
snoRNAs: small nucleolar RNAs
snRNAs: small nuclear RNAs
TARGET: therapeutically applicable research to generate effective treatments
TCGA: the cancer genome atlas
TNBC: triple‐negative breast cancers
WGCNA: weighted gene correlation network analysis

## 1 Introduction

Breast cancer (BC) is the second leading cause of cancer incidence worldwide, accounting for 11.6% of all cancer cases [1]. It ranges from noninvasive to highly aggressive invasive forms, with each subtype defined by distinct histological and molecular characteristics that shape clinical outcomes and guide treatment strategies [2, 3, 4]. Ductal carcinoma *in situ* (DCIS) is characterized by abnormal epithelial proliferation limited within the basement membrane. A subset of DCIS cases may progress to invasive disease [5, 6]. Invasive ductal carcinoma (IDC) constitutes approximately 70–80% of invasive breast cancers and is defined by cohesive nests or cords of malignant cells infiltrating the stroma [7]. In contrast, invasive lobular carcinoma (ILC), accounting for 10–15% of invasive BC, features a hallmark loss of E‐cadherin, leading to dispersed, single‐file cell infiltration and often poses diagnostic challenges during imaging [8]. Molecular classification, determined by immunohistochemical marker expression profiles, delineates four principal intrinsic subtypes of BC. Luminal A (Lum‐A) tumors are hormone receptor positive (HR+), express estrogen receptor (ER) and/or progesterone receptor (PR), are human epidermal growth factor receptor 2 (HER2) negative (HER2‐), and exhibit low Ki‐67 proliferation indices. These tumors typically follow a slow progression trajectory and respond favorably to endocrine therapy. Luminal B (Lum‐B) tumors are also HR+ but display higher Ki‐67 proliferation rates and may overexpress HER2. This biological profile is associated with a higher risk of relapse, often necessitating combined chemo‐endocrine therapy to optimize clinical outcomes [9]. HER2 BC subtype is characterized by HER2 overexpression. Although historically considered highly aggressive, the introduction of HER2‐targeted therapies has dramatically improved its prognosis, with early‐stage patients achieving over 90% 5‐year disease‐free survival rates [10]. Triple‐negative breast cancers (TNBC), lacking ER, PR, and HER2, are the most aggressive subtype, with high proliferative indices, poor prognosis, and limited treatment options [11]. Thus, integrating histopathological and molecular subtyping is essential for precise prognostication and treatment planning in BC.

BC screening methods, such as self‐examinations and mammography, have significantly contributed to improved detection rates [1, 12]. Additionally, increased awareness, early diagnosis, and advancements in treatment have collectively helped reduce BC‐related mortality, particularly in developed regions [13]. Beyond mammography, advancements in molecular diagnostics, ranging from immunohistochemical panels to next‐generation sequencing, have revolutionized the identification of genetic and molecular markers in BC. These advancements have not only enhanced diagnostic precision but also improved risk assessment, recurrence prediction, and personalization of treatment strategies. Molecular diagnostics are indispensable for identifying markers of BC, such as HER2 overexpression, which correlates with aggressive cancer, increased metastatic potential, and elevated Ki‐67 levels, which reflect heightened cell proliferation. These markers provide critical insights for personalized therapy and optimized disease management. However, the utility of many biomarkers and genomic assays is constrained by tumor heterogeneity among patients, limiting their effectiveness to specific subgroups. For example, among multigene assays, Oncotype DX, MammaPrint, and the Breast Cancer Index have become integral to the management of early‐stage, HR+, HER2‐ breast cancers. These tests refine prognostic evaluation and guide chemotherapy benefit for lymph node‐negative tumors and, in selected postmenopausal patients, those with lymph node 1–3 positive disease and a T1–T2 primary tumor (≤ 5 cm), as demonstrated in the MINDACT trial [14]. Current NCCN and ASCO guidelines do not support routine use of MammaPrint in HER2+ or TNBC; evidence in these subtypes remains exploratory. The integration of these molecular tests into clinical practice underscores the importance of molecular insights in tailoring interventions and improving outcomes for BC patients [15]. However, their application in lower‐income regions and community hospitals remains restricted due to infrastructural deficits and lack of specialized medical staff in incorporating these diagnostics into clinical routines. Therefore, there is a pressing need for a globally accessible, less invasive molecular biomarker assay that can identify BC invasiveness and metastasis while facilitating ongoing health monitoring following treatment [16]. BC diagnostics traditionally rely on imaging, primarily mammography along with ultrasound (echography), *in situ* biopsy, and anatomo‐pathological analysis of solid tumor tissue. However, these procedures are often associated with discomfort and potential complications. Recently, saliva has emerged as a promising alternative for BC screening, diagnosis, and monitoring [17]. Saliva‐based diagnostics use a variety of biomarkers, including proteomic, metabolomic, and transcriptomic profiles to identify cancer markers [18]. Unlike blood, saliva collection is noninvasive, causing minimal discomfort and offering greater patient acceptability and accessibility. Previous studies have highlighted the diagnostic potential of salivary biomarkers, such as CA15‐3 (MUC1) and HER2 proteins, to differentiate BC patients from healthy individuals [19, 20]. Additionally, RNA markers, like *KCNJ3* mRNA and *MIR‐21*, have shown promise as BC indicators in specific populations [18, 21, 22, 23]. Expanding this research to include the entire RNA spectrum in saliva could further enhance the sensitivity and specificity of diagnostics, potentially revealing additional RNA species and interactions linked to BC subtypes or disease progression. In this context, a thorough and clinically significant analysis of the salivary profile in BC patients is currently lacking and could yield critical insights for the advancement of diagnostic and prognostic applications in this field.

This study presents a comprehensive analysis of the salivary transcriptome in BC patients, revealing distinct RNA expression patterns associated with disease subtypes and progression, and potential for noninvasive molecular diagnostics. Using whole‐transcriptome RNA sequencing, we analyzed a diverse array of RNA species, including coding mRNAs, long noncoding RNAs (lncRNAs), microRNAs (miRNAs), and small nuclear RNAs (snRNAs) that together reflect key features of BC biology. Several genes were evenly expressed across both cancer and control groups, providing potential reference markers for future salivary RNA research. Notably, we identified RNA signatures that were differentially expressed relative to control samples, enabling the identification of both histological and HR+ molecular subtypes. Additionally, expression patterns linked to lymph node involvement were also detected. Importantly, concordance between these salivary markers and tumor‐derived profiles supports their potential for noninvasive molecular stratification. Specific salivary genes exhibited correlations with poor clinical outcomes, suggesting prognostic relevance for identifying high‐risk patients. In a small follow‐up subset, modest changes in selected RNA markers were observed 1‐year posttreatment, suggesting potential for monitoring disease and treatment dynamics over time.

This proof‐of‐concept study not only highlights the diagnostic, prognostic, and treatment follow‐up utility of salivary RNA but also offers a noninvasive approach for BC detection and real‐time monitoring. Although conducted on a restricted cohort, the results showed a high level of significance for the identified RNA signatures, along with a strong correlation to established BC hallmarks. These findings warrant further validation on a larger clinical cohort to pave the way for broader clinical application.

## 2 Materials and methods

### 2.1 Ethical approval and patient consent

The saliva collection was conducted at the Multidisciplinary Breast Clinic, Antwerp University Hospital, following protocols approved by the Institutional Review Board and UZA clinical ethical committee under reference number (BB20079). The study methodologies conformed to the standards set by the Declaration of Helsinki. All participants provided informed consent prior to participation. Detailed instructions regarding the saliva collection procedure were explained to each participant to ensure proper understanding and compliance. Saliva samples from the original and independent cohort were collected between September 2022 and February 2025.

### 2.2 Saliva collection and processing

Unstimulated saliva samples were collected from BC patients and healthy individuals using DNA Genotek collection tubes (Ref CP‐190). Participants were instructed to avoid eating, drinking, smoking, or oral hygiene activities for at least 30 min before collection to reduce variability in saliva composition. Each saliva‐filled tube was shaken vigorously for at least 8 s to ensure sample homogeneity. Samples were then transported to the UZA Biobank, where they were further processed. In their original collection vials, samples were incubated at 50 °C for 1 h in a water bath. After incubation, samples were aliquoted into 1 mL portions in separate microcentrifuge tubes for storage at −80 °C. Saliva samples were thawed on ice, and a 0.5 mL aliquot was incubated at 90 °C for 15 min to inactivate RNases, then cooled to room temperature. A neutralizer solution (20 μL, 1 : 25) was added to the saliva aliquot, followed by a 10‐min incubation on ice. Samples were then centrifuged at maximum speed for 3 min. The supernatant was carefully transferred to a fresh 5‐mL tube without disturbing the pellet. The samples were then stored at −80 awaiting further RNA extraction.

### 2.3 Patient cohorts

The study included multiple cohorts to evaluate the utility of salivary RNA biomarkers for BC diagnosis and monitoring. Detailed cohort demographics and sample characteristics are provided in Table S1. The main prospective cohort comprised 40 BC patients (*n* = 40) and 10 healthy controls (Ctrl; *n* = 10), recruited at the time of initial mammography. An independent validation cohort (BC = 20, Ctrl = 12), collected later within the same diagnostic time frame, included 15 IDC and 5 ILC cases, with molecular subtypes of 6 Lum‐A and 14 Lum‐B. A follow‐up cohort included 15 paired samples collected ~ 1 year after baseline from participants in the prospective cohort. All cohorts were recruited in accordance with ethical guidelines, with informed consent obtained from all participants.

### 2.4 Immunohistochemical and molecular assessment of tumor biomarkers and lymph node status

Immunohistochemistry (IHC) was performed on formalin‐fixed, paraffin‐embedded (FFPE) tissue to evaluate ER, PR, Ki‐67, and HER2 in cases of invasive BC. ER and PR positivity was defined as nuclear staining in ≥ 1% of tumor cells. Ki‐67 was scored as a percentage of positively stained tumor nuclei and categorized into low (≤ 15%) or high (≥ 15%) proliferation. HER2 status was assessed via IHC, and cases with 2+ (equivocal) or 3+ (positive) staining underwent reflex testing by fluorescence *in situ* hybridization (FISH). FISH was also selectively performed for 1+ cases when staining was ambiguous and was conducted at the pathologist's discretion. Samples that tested negative by FISH were considered HER2 negative. HER2 testing was not routinely performed in cases of DCIS. LN status was determined via sentinel lymph node biopsy (SLNB), with histopathological assessment and cytokeratin IHC when necessary. Positive SLNB findings were followed by further axillary dissection according to clinical guidelines.

### 2.5 RNA isolation from cell‐free saliva

RNA was isolated from cell‐free saliva samples using the miRNeasy Serum/Plasma Advanced Kit (Cat no.: 217184; Qiagen), following the manufacturer's protocol with slight modifications. To each sample, 2.5 mL of QIAzol lysis reagent was added, and the mixture was vortexed to ensure thorough lysis, followed by a 5‐min incubation at room temperature. Chloroform (500 μL) was added to the tubes and vortexed for 15 s, incubated at room temperature for 2–3 min, and centrifuged at 12 000 ***g*** at 4 °C for 15 min. The upper aqueous phase was carefully transferred to a new 5 mL tube, avoiding contamination from the interphase, and mixed with 1.5 volumes of 100% ethanol (final volume ~ 5 mL). For RNA purification, the sample was loaded in 700 μL aliquots onto an RNeasy MinElute spin column connected to a Vac‐Man® Laboratory Vacuum Manifold. The vacuum was opened carefully. The process was repeated until the entire sample was loaded. The column was then removed from the vacuum manifold, placed in a 2‐mL collection tube, and washed with 700 μL Buffer RWT, followed by centrifugation at ≥ 8000 ***g*** for 15 s. The column was subsequently washed with 500 μL Buffer RPE and centrifuged at ≥ 8000 ***g*** for 15 s, followed by a final wash with 500 μL of 80% ethanol and centrifugation at ≥ 8000 ***g*** for 2 min at room temperature. The column was transferred to a fresh 2 mL collection tube with the lid open and centrifuged at full speed for 5 min to dry the membrane. Finally, the column was placed into a new 1.5 mL collection tube, and RNA was eluted by applying 30 μL of RNase‐free water directly to the center of the membrane, followed by centrifugation at full speed for 1 min. RNA was initially isolated from saliva samples, and its integrity was evaluated using the Agilent Bioanalyzer 2100 System (Agilent Technologies, United States) prior to submission to the sequencing service. The eluted RNA was stored at −80 °C for subsequent downstream analyses.

### 2.6 RNA sequencing

RNA sequencing was performed at Novogene (Cambridge, United Kingdom). First‐strand cDNA synthesis was carried out using random hexamer primers, followed by second‐strand cDNA synthesis using either dUTP for directional libraries or dTTP for nondirectional libraries. Library quality was assessed using Qubit for quantification, real‐time PCR, and a bioanalyzer for size distribution analysis. Quantified libraries were pooled and sequenced on Illumina platforms with a single‐end read length of 50 base pairs. Sequencing was conducted without mRNA selection, ensuring an unbiased representation of all RNA species present in the samples.

### 2.7 RNA‐seq data analysis

RNA‐seq data preprocessing was performed using the nf‐core/rnaseq pipeline [24], version 3.12.0, with default settings except for the following modifications: (a) To remove bacterial reads, read filtering was applied by referencing bacterial genomes from the Human Oral Microbiome Database (HOMD, https://www.homd.org/, accessed on 2024‐02‐21). Specifically, the largest genome for each relevant bacterial species was retrieved from HOMD, compiled into a multi‐fasta file, and incorporated using the ‘‐‐bbsplit_index’ parameter in nf‐core for filtering, with genome identifiers provided in Table S2. (b) The Ensembl GRCh38 release 109 was employed as the reference human genome for aligning human RNA reads. (c) To account for GC content bias, the quantification step was run with Salmon using the ‘—gcBias’ parameter. Following preprocessing, the resulting count file was used as input for differential expression analysis (DEA), which was performed with DESeq2 (version 1.38.3) on R (version 4.2.2) to allow for robust identification of differentially expressed genes. Preliminary analyses, including quality assessments and exploratory data visualizations, were conducted using R scripts from the iDEP package [25].

### 2.8 Stable gene analysis in saliva

To identify genes with stable expression across saliva samples, a preliminary filtering criterion was applied whereby only genes with a normalized expression value of at least 1 across all 40 BC samples and 10 control (Ctrl) samples were retained for analysis. This initial selection yielded 3048 genes consistently expressed across both BC and Ctrl samples, which were subsequently included in the stability analysis. Stability of gene expression was assessed using two complementary methods. First, gene stability was evaluated with the geNorm algorithm [26], which assigns an ‘M’ value to quantify stability based on pairwise variation across samples, with lower M values indicating higher stability. In parallel, the coefficient of variation (CV) of expression was calculated for each gene to capture the relative variability in expression levels [27]. Based on the rankings obtained from both the geNorm M value and CV, a cumulative rank score was calculated to prioritize genes exhibiting the most stable expression across all samples. Genes with the lowest cumulative ranks were considered the most stably expressed and suitable for normalization or reference purposes in downstream analyses.

### 2.9 Co‐expression network analysis

Co‐expression network analysis was conducted on normalized gene expression values from the BC cohort using Weighted Gene Correlation Network Analysis (WGCNA) implemented in the WGCNA R package [28]. WGCNA allows the identification of clusters, or modules, of highly correlated genes, providing insights into gene co‐expression patterns within the dataset. Modules were summarized by their module eigengenes, representing the primary expression pattern within each cluster, and intramodular hub genes, which serve as central nodes within modules. Additionally, eigengene network methodology was applied to examine relationships between modules and external sample traits. Using a dynamic tree cut dendrogram, the analysis identified four distinct co‐expressed gene groups, each assigned as separate modules, reflecting underlying patterns of co‐regulation among genes in the BC cohort.

### 2.10 Cancer hallmark and EMT gene analysis

DE genes identified in this study were analyzed for their association with cancer hallmarks by comparing them against the ‘Integrated Cancer Hallmark Gene Set’, a curated database of 6763 candidate genes associated with cancer. This comprehensive gene set was compiled by integrating and merging gene data from seven established resources to cover a wide spectrum of cancer‐related genes [29]. Assessment of gene involvement in EMT processes was performed by identifying overlaps with datasets available in the EMTome database (http://www.emtome.org/) [30].

### 2.11 Expression analysis of selected signatures in control, tumor, and metastasis

The tnm plot tool (https://www.tnmplot.com/) was used to evaluate the expression of salivary signatures identified through DE analysis across control, tumor, and metastatic samples. Violin and density plots, along with related statistical data for selected salivary signatures across these tissue types, were generated using this tool. Gene expression analysis in tumor, normal, and metastatic tissues utilized data from the TNM plot, which includes 56 938 samples: 33520 samples from 3180 gene chip‐based studies (453 metastatic, 29 376 tumorous, and 3691 normal samples), 11 010 samples from TCGA (394 metastatic, 9886 tumorous, and 730 normal samples), 1193 samples from therapeutically applicable research to generate effective treatments (TARGET) database (1 metastatic, 1180 tumorous, and 12 normal samples), and 11 215 normal samples from GTEx. [31]. Further subtype/condition‐specific tumor expression patterns of selected salivary genes (such as comparisons between IDC vs ILC, Lum‐A vs Lum‐B, and LN+ vs LN−) were analyzed using the Breast Cancer Gene‐Expression Miner v5.2 (bc‐GenExMinerv 5.2) platform (https://bcgenex.ico.unicancer.fr/BC‐GEM/GEM‐requete.php) [32]. Targeted expression analysis was conducted to visually compare gene expression between patient groups based on specific clinical or molecular criteria. Expression levels were derived from RNA‐seq data, incorporating The Cancer Genome Atlas (TCGA) and Scan‐B datasets to ensure comprehensive coverage of BC subtypes and pathological features.

### 2.12 Signature‐based survival analysis

Survival analysis of overexpressed salivary RNA biomarkers was conducted using the Kaplan–Meier Plotter tool (https://kmplot.com/analysis/) [33], which integrates gene expression data from both microarray and RNA‐seq platforms across a large cohort of BC patients. For each gene of interest, overall survival (OS) probabilities were assessed. Subtype‐specific analyses were performed by utilizing the ‘Restrict analysis to subtypes…’ feature. This approach enabled the evaluation of prognostic significance within distinct BC subgroups.

### 2.13 Statistical testing

Statistical analysis for DE was conducted using the DESeq2 package in R, which uses a model based on the negative binomial distribution to account for the variability and count‐based nature of RNA‐seq data. Significance of differential expression is determined by the Wald test for each gene, and *P*‐values are adjusted for multiple testing using the Benjamini–Hochberg method to control the false discovery rate (FDR). Genes with an adjusted *P*‐value (FDR) below 0.05 and log_2_FC > |2|, were considered significantly differentially expressed. For expression analysis with the TNM plot tool, statistical significance was computed using Kruskal–Wallis tests to compare gene expression across control, tumor, and metastatic tissues, with FDR correction applied using the Benjamini–Hochberg method. In quantitative reverse transcription PCR (qRT‐PCR) experiments, statistical significance was assessed using a two‐tailed Mann–Whitney *U*‐test. Significance thresholds were set at *P* < 0.05 (indicated by *), *P* < 0.005 (indicated by **) and *P* < 0.0005 (indicated by ***).

### 2.14 Quantitative reverse transcription PCR (qRT‐PCR) validation and analysis

qRT‐PCR was conducted to assess the expression of selected genes in saliva samples. Primers were specifically designed to amplify all isoforms of each target gene, ensuring comprehensive detection of gene expression across multiple transcript variants present in the saliva. qRT‐PCR reactions were set up with SYBR Green Master Mix (Qiagen) and run on a qTOWER Real‐Time PCR Thermal Cycler. Relative expression levels were calculated using the ΔΔCt method, with genes selected based on prior stability analysis in saliva. Each sample was run in duplicate, and no‐template controls were included to verify the absence of contamination. The list of primers used in this study can be found in Table S3.

Candidate genes for qRT‐PCR validation in an independent cohort were selected from the transcriptomic data using distinct criteria for both potential biomarkers and stable reference genes. Potential biomarkers, including those for BC and specific histological and molecular subtypes, were prioritized from the list of upregulated genes based on a combination of factors, namely: high statistical significance (FDR < 0.05), substantial log_2_ fold change, concordant expression profiles in public tumor datasets, and established biological relevance to oncogenic processes. The selection of stable reference genes for validation was chosen based on two additional essential criteria: their moderate‐to‐high expression levels in the RNA‐seq data, which ensures reliable and reproducible detection in qRT‐PCR assays, and sequence characteristics amenable to the design of specific, high‐efficiency primers that span exon‐exon junctions.

## 3 Results

### 3.1 Study outline and patient characteristics

**Fig. 1:**
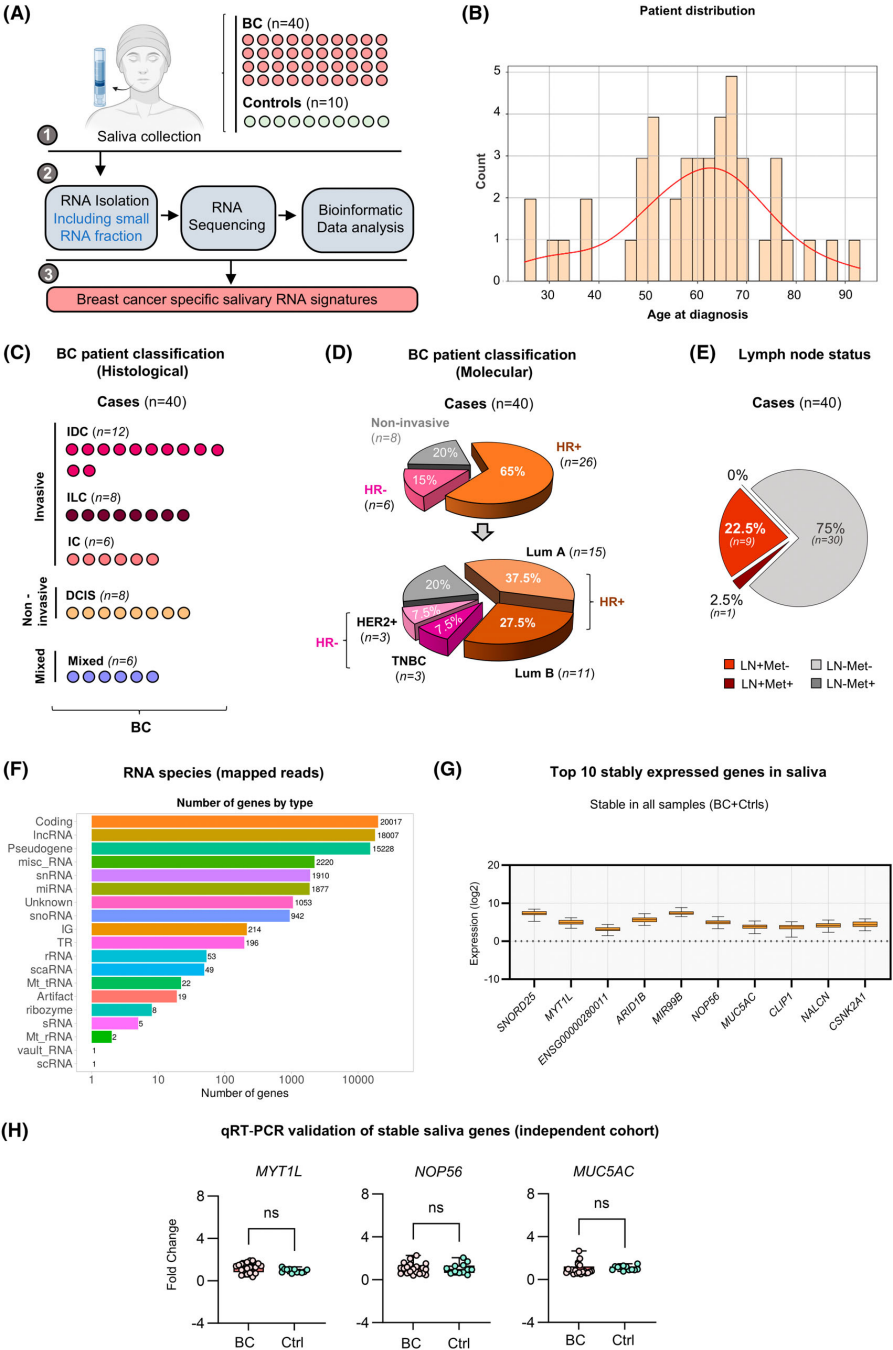
Study design and analysis of salivary RNA profiles in breast cancer (BC) patients and controls. (A) Schematic representation of the experimental workflow. Saliva was collected from BC patients (BC, *n* = 40) and healthy controls (Ctrls, *n* = 10). RNA isolation, including the small RNA fraction, RNA sequencing, and bioinformatic analysis, was performed to identify breast cancer‐specific salivary RNA signatures. Patient illustration was generated using BioRender.com. (B) Histogram showing the age distribution of BC patients at diagnosis, ranging from 25 to 93 years. A density curve is overlaid to visualize the trend, with a peak between 50 and 70 years. (C) Histological classification of BC patients (*n* = 40). Patients were categorized into invasive ductal carcinoma (IDC, *n* = 12), invasive lobular carcinoma (ILC, *n* = 8), invasive carcinoma not otherwise specified (IC, *n* = 6), mixed histological features (*n* = 6), and ductal carcinoma *in situ* (DCIS, *n* = 8). Each dot in the figure represents an individual patient. (D) Molecular classification of BC patients. Among the 40 BC patients, 65% (*n* = 26) were hormone receptor‐positive (HR+), further divided into Luminal A (*n* = 15, 37.5%) and Luminal B (*n* = 11, 27.5%) subtypes. The remaining 15% (*n* = 6) were hormone receptor‐negative (HR−), with human epidermal growth factor receptor 2‐positive (HER2+) (*n* = 3, 7.5%) and triple‐negative BC (TNBC, *n* = 3, 7.5%) cases identified. The rest 20% (*n* = 8) represented the noninvasive fraction. (E) Pie chart illustrating lymph node involvement and metastatic status were also assessed. Among 40 BC patients, the groups showing lymph node (LN) spread or metastasis (Met) were: LN+Met‐: 22.5% (*n* = 9); LN+Met+: 2.5% (*n* = 1). LN‐Met+ cases were absent (0%, *n* = 0), while LN‐Met‐: 75% (*n* = 30). (F) Bar graph depicting the types of RNA species identified from mapped reads in saliva. (G) Box plots showing expression of the top 10 stably expressed genes in saliva identified across all 50 samples (Ctrl and BC) cohorts. Whiskers represent the full range from minimum to maximum values (H) qRT‐PCR validation of selected stable genes (*MYT1L, NOP56, and MUC5AC*) in an independent cohort of Ctrl (*n* = 12 patients) and BC (*n* = 20 patients) samples. Fold change denotes RNA expression relative to Ctrl, plotted as log_2_(fold change). Error bars indicate standard error of the mean (SEM). Statistical significance was assessed using a two‐tailed Mann–Whitney *U*‐test; no significant differences (ns) in expression were observed between the two groups.

In this study, the saliva transcriptome of 40 BC patients and 10 healthy individuals (Ctrls) was evaluated. Total RNA, including the small RNA fraction, was extracted from the saliva samples collected during initial mammography screening, then sequenced and bioinformatically analyzed to identify BC‐specific RNA signatures and underlying subtypes (Fig. 1A). The BC group average age was 59.7 years (range: 25–93 years) at diagnosis, older than the average of the Ctrl group at 51.1 years (range: 32–68 years) (Fig. 1B and Table S1). For this study, the BC types IDC, ILC, and DCIS were included in the analysis. BC patients were categorized according to histological types: 30% (*n* = 12) had IDC, 20% (*n* = 8) had ILC, and 15% (*n* = 6) had invasive carcinomas that could not be categorically identified as either IDC or ILC. Additionally, 20% (*n* = 8) of patients were diagnosed with DCIS, representing the noninvasive BC group [34, 35], while 15% (*n* = 6) had mixed histological features, combining both noninvasive and invasive BC components (Fig. 1C).

Molecular classification was performed based on HR status, HER2 expression, and the Ki‐67 proliferation index. Among the cohort, 65% (*n* = 26) were HR+. These HR+ cases were further stratified by Ki‐67 index into Lum‐A (37.5%, *n* = 15) with low Ki‐67 (< 15%) and Lum‐B (27.5%, *n* = 11) with high Ki‐67 (> 15%) subtypes (Fig. 1D, Fig. S1 and Table S1). Additionally, 15% (*n* = 6) of patients had HR‐ tumors, including HER2+ (7.5%, *n* = 3) and TNBC (7.5%, *n* = 3) cases (Fig. 1D, Fig. S1 and Table S1). HER2‐enriched tumors exhibited high HER2 expression with low‐to‐moderate PR and ER levels and a Ki‐67 index averaging 15%. TNBCs lacking ER, PR, and HER2 expression were marked by a high Ki‐67 proliferative index, reflecting rapid tumor proliferation and a clinically aggressive course (Fig. S1). Given their higher prevalence in the study population, our analysis primarily focused on the HR+ molecular subgroup, with particular emphasis on the comparative characteristics of the Lum‐A and Lum‐B subtypes. Furthermore, lymph node spread and metastatic status were assessed in the 40 BC patients. Among them, 22.5% (*n* = 9) had lymph node involvement without distant metastasis (LN+Met−), indicating regional disease spread. A single patient (2.5%) exhibited lymph node involvement with distant metastases (LN+Met+). The majority of cases, 75% (*n* = 30), exhibited neither lymph node involvement nor reported distant metastasis (LN‐Met‐), suggesting early‐stage disease (Fig. 1E). Clinical and pathological data for all patients and controls are summarized in Table S1.

### 3.2 Identification and validation of stably expressed salivary RNA for BC biomarker studies

RNA sequencing of saliva from 40 BC patients and 10 controls (Ctrls), following bacterial read removal, revealed diverse human RNA species, including coding RNAs, lncRNAs, snRNAs, and miRNAs. Other species, like small nucleolar RNAs (snoRNAs), mitochondrial RNAs (mt‐RNAs), and catalytic RNAs, such as ribozymes, were also identified, underscoring the complexity of the salivary transcriptome (Fig. 1F).

We first focused on identifying stably expressed RNAs across all samples to establish reliable references. For this analysis, we have identified genes that showed consistent expression, with a minimum normalized expression value of at least ‘1’ across both BC and Ctrl samples. A total of 3048 genes were identified (Table S4). Among these, genes such as *SNORD25, MYT1L, MIR99B, NOP56,* and *MUC5AC* were consistently ranked among the top 10 most stably expressed genes across both BC and control groups. Their minimal expression variability suggests strong potential as reference genes for normalization in future studies (Fig. 1G, Table S5).

Validation using qRT‐PCR on an independent cohort (20 BC patients, 12 Controls) confirmed the stable expression of *MYT1L*, *NOP56*, and *MUC5AC* between the BC and Ctrl groups, establishing their reliability as internal reference genes (Fig. 1H).

### 3.3 Salivary RNA signatures for BC detection and histological subtype characterization

**Fig. 2:**
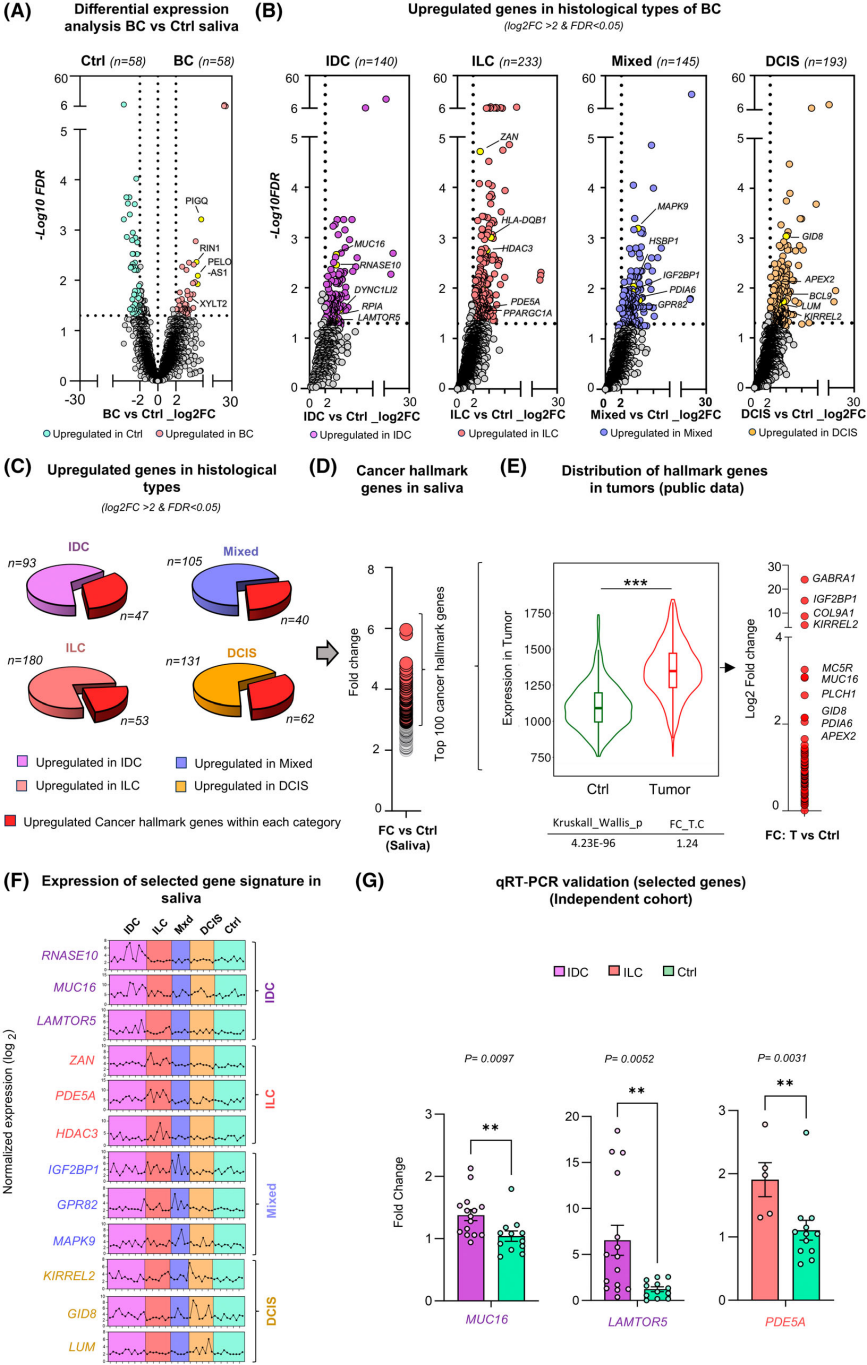
Differential expression analysis of salivary RNA across breast cancer (BC) and its histological types. (A) volcano plot illustrates differentially expressed genes (DEGs) in saliva samples from BC patients, compared with healthy controls (Ctrl). The x‐axis represents the log_2_ fold change (log_2_FC) in gene expression between BC and Ctrl samples, while the y‐axis represents the ‐log10 false discovery rate (FDR). Genes significantly upregulated in BC samples (*n* = 58) with a log_2_FC > 2 and FDR < 0.05 are highlighted in red. Genes significantly upregulated in Ctrl samples (*n* = 58) are highlighted in green, while nonsignificant genes are shown in gray. (B) Volcano plots display upregulated genes in saliva samples from patients with invasive ductal carcinoma (IDC, *n* = 140), invasive lobular carcinoma (ILC, *n* = 233), mixed condition (*n* = 145), and ductal carcinoma *in situ* (DCIS, *n* = 193) compared with healthy controls. The x‐axis represents the log_2_FC in gene expression between each subtype and controls, while the y‐axis represents the −log10 FDR. Significant upregulated genes (log_2_FC > 2, FDR < 0.05) are highlighted, with key cancer hallmark genes labeled for each subtype. (C) Pie charts depict the proportion of cancer hallmark genes among upregulated genes in each histological type: IDC (47/140), ILC (53/233), mixed carcinoma (40/145), and DCIS (62/193), emphasizing their distribution within different BC histological types. (D) A scatter plot showing the fold change (FC) of the top 100 cancer hallmark genes upregulated in saliva of histological types of BC. (E) A violin plot comparing the expression levels of hallmark genes in tumors versus controls using public tumor expression data, indicating a highly significant difference (fold change tumor vs. control (FC_T.C) = 1.24, Kruskal–Wallis *P* = 4.32e‐96). Significance is denoted by *** (*P* < 0.0005). (Right) A corresponding log_2_FC plot highlights key upregulated hallmark genes in tumors. (F) Salivary expression profiles of the cancer hallmark genes, selected from the top 100 upregulated hallmark genes across BC histological types (IDC, ILC, Mixed & DCIS) over control samples. Points connected by lines represent normalized expression values (log_2_) for individual samples, with variability observed within each group. (G) Validation of selected salivary upregulated genes in an independent cohort. Fold change denotes RNA expression relative to Ctrl, plotted as log_2_(fold change). Error bars indicate standard error of the mean (SEM). Statistical significance was assessed using a two‐tailed Mann–Whitney *U*‐test, with exact *P*‐values of 0.0097 for *MUC16*, 0.0052 for *LAMTOR* *5*, and 0.0031 for *PDE5A*. Significance is indicated by * (*P* < 0.05) and ** (*P* < 0.005).

Differential expression analysis of salivary RNA sequencing data from 40 BC patients and 10 healthy Ctrls (Fig. S2A) identified 58 genes significantly upregulated in BC (log_2_ fold change (log_2_FC) > 2, FDR < 0.05), with a comparable number of genes also upregulated in the control group (Fig. 2A and Table S6). This robust list based on stringent threshold criteria provided a strong basis for identifying potential biomarkers for BC diagnosis. The top 10 upregulated genes in BC cover a diverse range of RNA species, including microRNAs (*MIR197*), noncoding RNAs (*PELO‐AS1* and *CIBAR1‐DT*), and coding genes (*TPRN*, *ASF1A*, *XYLT2*, *TMEM145*, *PIGQ*, and *RIN1*) (Fig. S2B). Several genes, like *ASF1A*, linked to chromatin assembly, gene regulation, and immune responses [36], and *RIN1*, a protein involved in endocytosis and cytoskeletal remodeling, have links to cancer progression [37, 38, 39]. Noncoding RNAs like *PELO‐AS1* and *CIBAR1‐DT* are also emerging as important regulators in cancer [40, 41]. Validation of some of the identified genes in an independent cohort corroborated the RNA‐seq findings (Fig. S2C), supporting their potential as robust BC biomarkers.

Co‐expression analysis of genes expressed in the BC patient pool revealed four distinct co‐expression modules: teal (MicroRNA Module, including *MIR374A*, *MIR425*, and *MIRLET7G* with potential involvement in cancer progression [42, 43, 44]), brown (SnoRNA Module, including *SNORD95*, *SNORD58*, and *SNORD121A*, some of which are implicated in cancer [45]), blue (Gene Regulation Module included both protein‐coding and noncoding genes, such as *LINC02901*, *MRPS12*, and *PSMC5*, associated with cellular structure and gene regulation [46, 47]) (Fig. S2D and Table S7), yellow (non‐coding RNA module includes *DPP10‐AS1* and *PDK1‐AS1* (LncIHAT), with potential regulatory roles in cancer [48, 49]) (Fig. S2D, Table S7). These modules consist of co‐regulated genes that show coordinated expression patterns linked to specific cellular processes, offering important insights into BC salivary RNA profiles.

Further analysis of DEGs, particularly upregulated ones across IDC, ILC, DCIS, and mixed BC cases identified 140 DEGs in IDC, 233 in ILC, 145 in mixed BC, and 193 upregulated DEGs in DCIS compared with controls, applying a stringent cutoff of log_2_FC > 2 and FDR < 0.05 (Fig. 2B and Table S8). To further characterize these upregulated genes, we assessed their association with core cancer‐related processes, such as proliferation, immune evasion, and genomic instability (recognized hallmarks of cancer), as well as key cellular programs like epithelial–mesenchymal transition (EMT). Comparative analysis using cancer hallmark and EMT‐related gene sets [29, 30] revealed that 202 upregulated genes across IDC, ILC, DCIS, and mixed BC subtypes were classified as hallmark genes, with an additional 22 genes specifically linked to EMT functions (Fig. 2C and Fig. S2E), underscoring their broader relevance to tumor biology. Notably, hallmark analysis identified 47 of the 140 significantly upregulated genes in IDC, 53 of 233 in ILC, 40 of 145 in mixed, and 62 of 193 in DCIS as key contributors to cancer hallmark processes (Fig. 2C and Table S8). Among these, the top 100 cancer hallmark genes upregulated in saliva across all BC subtypes (Fig. 2D) also demonstrated significant overexpression in tumor samples compared with controls, as assessed using TNM plot tool with data from The Cancer Genome Atlas (TCGA) and Therapeutically Applicable Research to Generate Effective Treatments (TARGET) databases (*n* = 11 066 tumor samples; *n* = 742 control samples) (Fig. 2E). This finding underscores that the salivary RNA signatures observed in BC patients reflect fundamental oncogenic processes, supporting their potential utility as diagnostic biomarkers.

Among the top 100 upregulated cancer hallmark genes, three representative genes per BC type were prioritized based on their upregulated expression in both tumor and saliva samples compared with the controls, leading to the selection of *KIRREL2*, *GID8*, and *LUM* for DCIS, *RNASE10*, *MUC16*, and *LAMTOR5* for IDC, *ZAN*, *PDE5A*, and *HDAC3* for ILC, and *IGF2BP1*, *GPR82*, and *MAPK9* for mixed forms (Fig. 2F). *RNASE10*, *MUC16*, and *LAMTOR5* were also selected as IDC markers due to their significantly higher expression in IDC compared with ILC tumors (TCGA, *n* = 549 vs. 116), providing further evidence of a stronger association with the IDC type (Fig. S2F). Likewise, *ZAN*, *PDE5A*, and *HDAC3* exhibited higher expression in ILC compared with IDC and were selected as ILC markers (Fig. S2F). qRT‐PCR validation of selected genes in an independent patient cohort demonstrated subtype‐related enrichment compared with controls (Fig. 2G), supporting the transcriptomic findings and highlighting these genes as potential histological type‐associated BC markers (Fig. 2G).

### 3.4 Salivary gene signatures associated with BC molecular subtypes

**Fig. 3:**
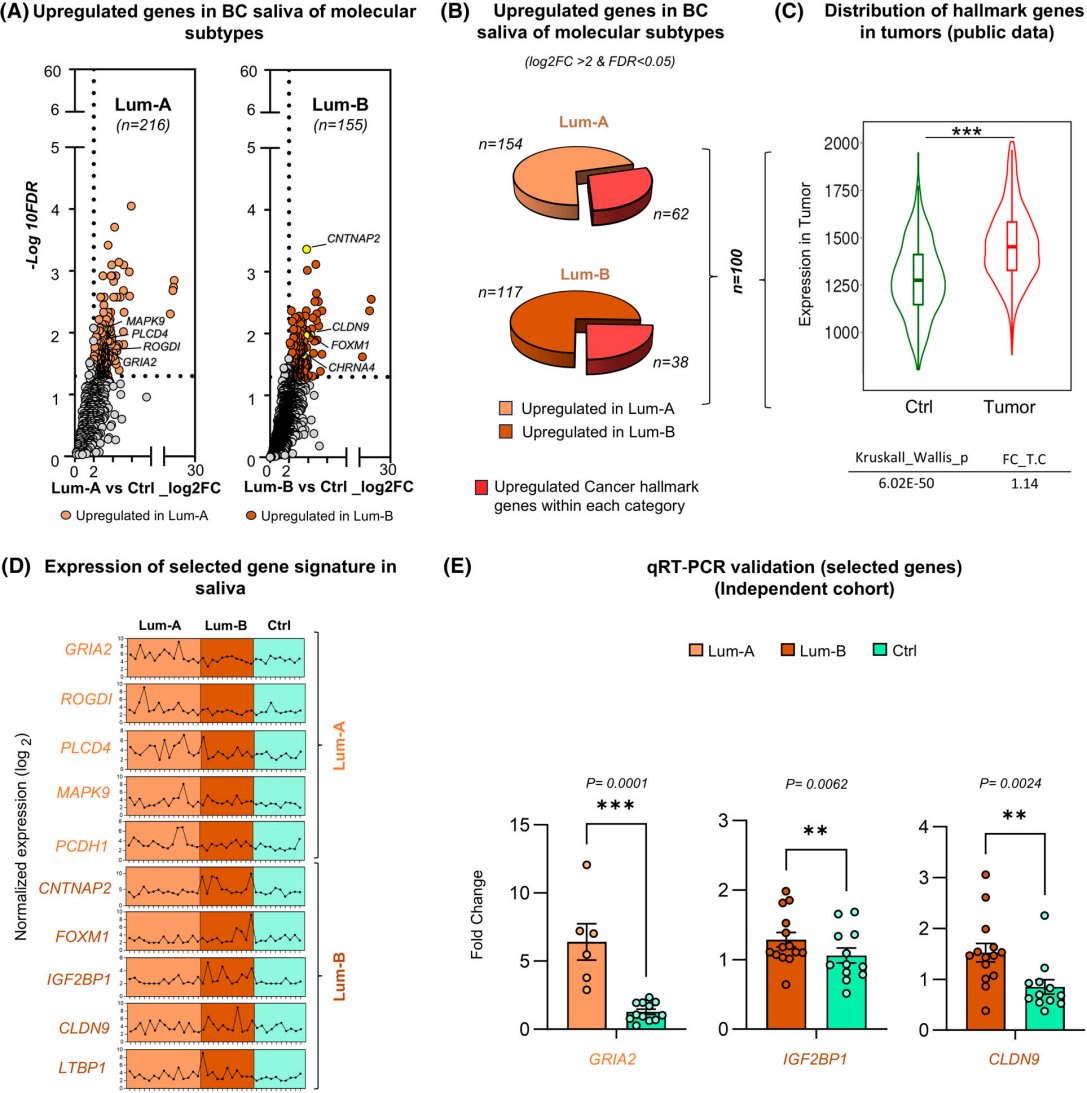
Differential expression analysis of salivary RNA in hormone receptor‐positive (HR+) breast cancer (BC) molecular subtypes. (A) Volcano plots show upregulated genes in saliva samples from HR+ BC molecular subtypes, specifically Luminal A (Lum‐A, *n* = 216 genes) and Luminal B (Lum‐B, *n* = 155 genes) compared with controls. The *x*‐axis represents the log_2_ fold change (log_2_FC) in gene expression, while the y‐axis represents the −log10 false discovery rate (FDR). (B) Genes with significant upregulation in each subtype (log_2_FC > 2, FDR < 0.05) are highlighted, and key cancer hallmark genes associated with each Luminal subtype are labeled. (B) Pie charts highlight the proportion of cancer hallmark genes identified among the DEGs in each molecular subtype, with 62 hallmark genes in Lum‐A and 38 in Lum‐B (total *n* = 100). (C) A violin plot comparing the expression levels of hallmark genes in tumors versus controls using public tumor expression data (fold change tumor vs. control (FC_T.C) = 1.14, Kruskal–Wallis *P* = 6.02e‐50), reinforcing their potential biological relevance in BC progression. Significance is denoted by *** (*P* < 0.0005). (D) Expression profiles of the cancer hallmark genes, selected from the 100 upregulated hallmark genes across Lum‐A and Lum‐B molecular subtypes over control samples (*n* = 62, Lum‐A and *n* = 38, Lum‐B). Points connected by lines represent normalized expression values (log 2) for individual samples, with variability observed within each group. (E) Validation of selected upregulated genes in independent cohort Lum‐A (*n* = 6 patients) and Lum‐B (*n* = 14 patients). Fold change denotes RNA expression relative to Ctrl, plotted as log2 (fold change). Error bars indicate standard error of the mean (SEM). Statistical significance was assessed using a two‐tailed Mann–Whitney *U*‐test, with *P*‐values of 0.0001 for *GRIA2*, 0.0062 for *IGF2BP1*, and 0.0024 for *CLDN9*. Significance is denoted by * (*P* < 0.05), ** (*P* < 0.005) and *** (*P* < 0.0005).

To characterize the molecular subtypes using salivary RNA, we focused specifically on HR+ BC patients (*n* = 26), as the hormone receptor‐negative (HR‐) subset (*n* = 6) was too limited for meaningful analysis. Within the HR+ group, our analysis centered on both Lum‐A and Lum‐B subtypes. DE analysis comparing Lum‐A and Lum‐B patients to controls revealed a higher number of upregulated genes in Lum‐A (*n* = 216) than in Lum‐B (*n* = 155) (log_2_FC > 2, FDR < 0.05), highlighting molecular differences in salivary RNA profiles (Fig. 3A and Table S9).

Further analysis revealed a total of 100 cancer hallmark genes, 62 in Lum‐A and 38 in Lum‐B subtype (Fig. 3B and Table S9), and 13 EMT‐related genes associated with these subtypes (Fig. S3A). Cross‐referencing the 100 cancer hallmark genes with TCGA and TARGET datasets using the TNM plot tool further revealed their overexpression in BC tumor samples compared with controls, emphasizing their role in oncogenic processes (Fig. 3C). Subtype‐associated hallmark genes were then selected based on their expression in both saliva and tumors, including *GRIA2*, *ROGDI*, *PLCD4*, *MAPK9*, and *PCDH1* for Lum‐A, and *CNTNAP2*, *FOXM1*, *IGF2BP1*, *CLDN9*, and *LTBP1* for Lum‐B (Fig. 3D). We identified hallmark genes linked to each subtype based on their expression in both saliva and tumor samples. For Lum‐A, key genes included *GRIA2*, *ROGDI*, *PLCD4*, *MAPK9*, and *PCDH1*; for Lum‐B, they included *CNTNAP2*, *FOXM1*, *IGF2BP1*, *CLDN9*, and *LTBP1* (Fig. 3D). These genes were also chosen because Lum‐A markers showed significantly higher expression in Lum‐A tumors (TCGA, *n* = 270) than in Lum‐B tumors (TCGA, *n* = 93), while Lum‐B markers were more highly expressed in Lum‐B tumors than in Lum‐A tumors, confirming their strong link to each subtype (Fig. S3B). Furthermore, qRT‐PCR validation of a subset of these genes in an independent cohort demonstrated luminal subtype‐specific overexpression relative to controls, further supporting their assignment as Lum‐A and Lum‐B markers (Fig. 3E). These findings support their relevance as potential molecular subtype‐specific salivary biomarkers.

### 3.5 Identification and validation of a metastatic salivary gene signature in BC

**Fig. 4:**
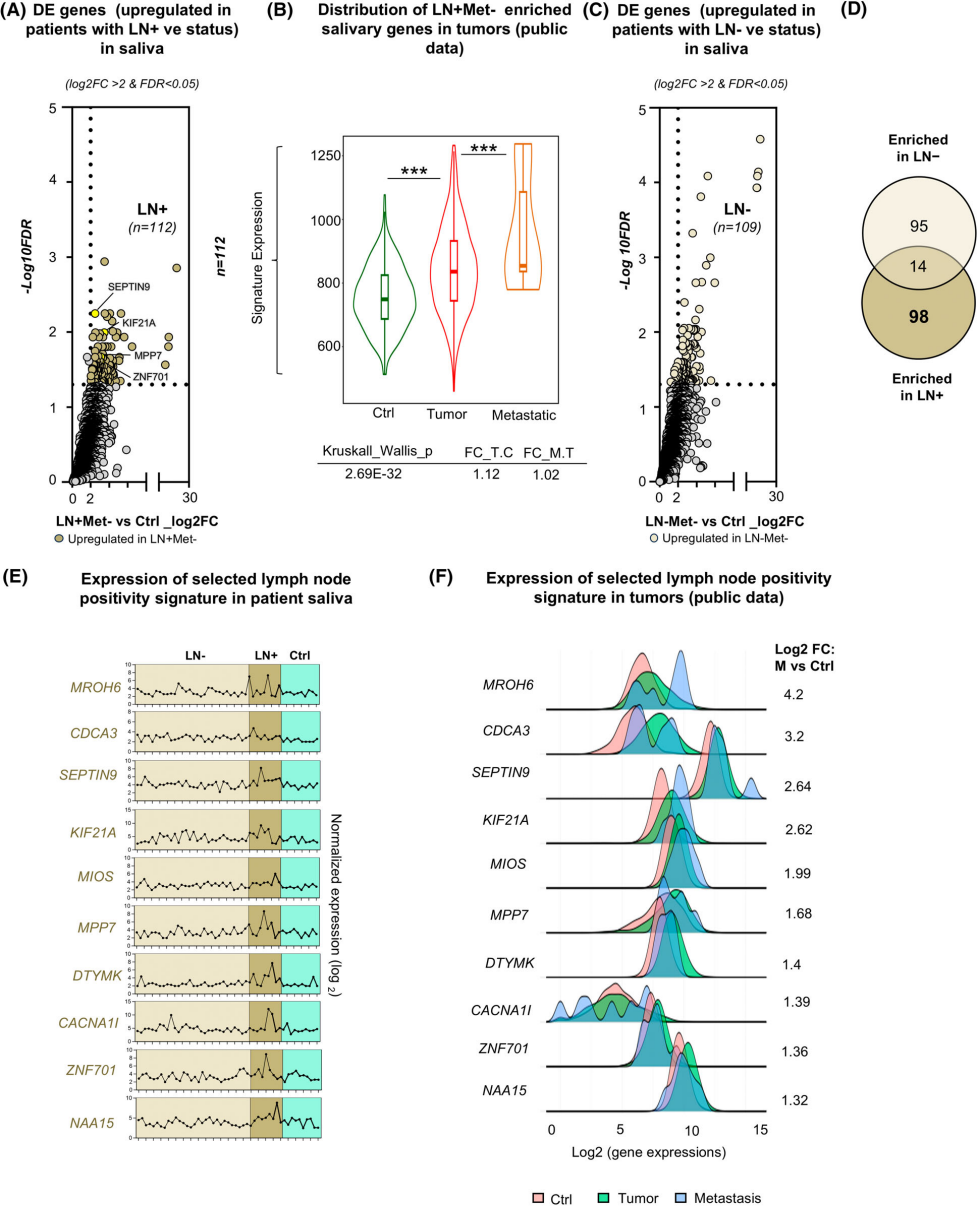
Differential expression analysis of salivary RNA associated with lymph node status in breast cancer (BC) patients. (A) Volcano plot displaying differentially upregulated genes in saliva from BC patients with lymph node‐positive (LN+) status (*n* = 112) compared with controls (Ctrl). The *x*‐axis represents the log_2_ fold change (log_2_FC) in gene expression, while the *y*‐axis represents the −log10 false discovery rate (FDR). Significantly upregulated genes in LN+ patients (log_2_FC > 2, FDR < 0.05) are highlighted, with key genes, such as SEPTIN9, KIF21A, MPP7, and ZNF701 labeled. (B) Violin plot comparing the expression levels of LN+Met‐ enriched salivary genes in tumors using public tumor expression data. The Kruskal–Wallis test indicates a highly significant difference (*P* = 2.69E‐32), with log_2_ fold changes of 1.12 (FC_T.C, Fold Change Tumor vs. Control) and 1.02 (FC_M.T, Fold Change Metastatic vs. Tumor), showing increased expression in tumors and metastatic cases. Significance is denoted by *** (*P* < 0.0005) (C) Volcano plot illustrating DEGs in saliva from BC patients with lymph node‐negative (LN−) status (*n* = 109) compared with controls. Genes significantly upregulated in LN− patients (log_2_FC > 2, FDR < 0.05) are highlighted. (D) Venn diagram showing the overlap between genes upregulated in LN+ and LN− patients. Fourteen genes were shared between both groups, suggesting common molecular signatures associated with lymph node status. (E) Expression profiles of genes specific to the LN+ group, selected from the 98 genes that were significantly upregulated in LN+ conditions compared with control saliva samples. Points connected by lines represent normalized expression values (log_2_) for individual samples, with variability observed within each group. (F) Density plots showing fold change (FC) values for selected saliva LN+ signature genes identified in public tumor datasets, comparing Ctrl, tumor, and metastasis samples.

To identify a salivary gene signature associated with lymph node metastasis in BC, the LN‐Met‐ (*n* = 30) and LN+Met‐ (*n* = 9) patient groups were analyzed based on the cohort composition. Only one patient had LN+Met+ status and none were in the LN–Met+ group; therefore, these groups were excluded from the study. DE analysis identified 112 significantly enriched genes in LN+Met‐ saliva compared with controls (log_2_FC > 2, FDR < 0.05) (Fig. 4A and Table S10). Since lymph node spread is often an early indicator of metastatic progression, cross‐analysis using public data to assess the 112 DE salivary genes confirmed overexpression of these genes in both tumor and metastatic cases compared with controls (Fig. 4B), highlighting their potential role in early metastatic transition. In contrast, 109 upregulated genes identified in the LN‐Met‐ group (Fig. 4C and Table S10) did not exhibit any increased expression in tumor or metastatic samples (Fig. S4A), suggesting that the salivary transcriptomic profile of LN‐Met‐ patients may be more reflective of localized disease rather than systemic progression. We then compared the DE genes enriched in LN‐Met‐ and LN+Met‐ cases, identifying 98 unique nonoverlapping genes specifically upregulated in the LN+ saliva (Fig. 4D and Table S10). Among these, 10 representative genes, including *MROH6*, *CDCA3*, *SEPTIN9*, *KIF21A*, *MIOS*, *MPP7*, *DTYMK*, *CACNA1I*, *ZNF701*, and *NAA15* (Fig. 4E) were selected based on their high expression levels in saliva and elevated expression in metastatic tumors compared with controls (Fig. 4F). Furthermore, these genes displayed significantly higher expression in tumors from LN+ metastatic cases (*n* = 1525) compared with LN‐ cases (*n* = 2345) (Fig. S4B), reinforcing their association with LN+ status. These findings reveal distinct salivary RNA signatures linked to lymph node involvement, indicating their potential utility as biomarkers for the detection of regional metastasis.

### 3.6 Identification of salivary RNA biomarkers correlated with BC prognosis

Building on data in this manuscript showing strong salivary–tumor gene‐expression concordance in BC patients, we investigated whether upregulated salivary gene signatures were linked to patient survival outcomes. Using public data, we assessed the survival relevance of 58 genes significantly enriched in BC patient saliva compared with healthy controls (Fig. 2A). Elevated expression of five genes *TMEM145*,*DGKZ*, *CHRNA4*, *CARHSP1*, and *ASF1A* were significantly associated with poor prognosis in a public clinical cohort of 2976 BC patients, with hazard ratios ranging from 1.52 to 1.83 and (FDR < 10%) (Fig. S5).

**Fig. 5:**
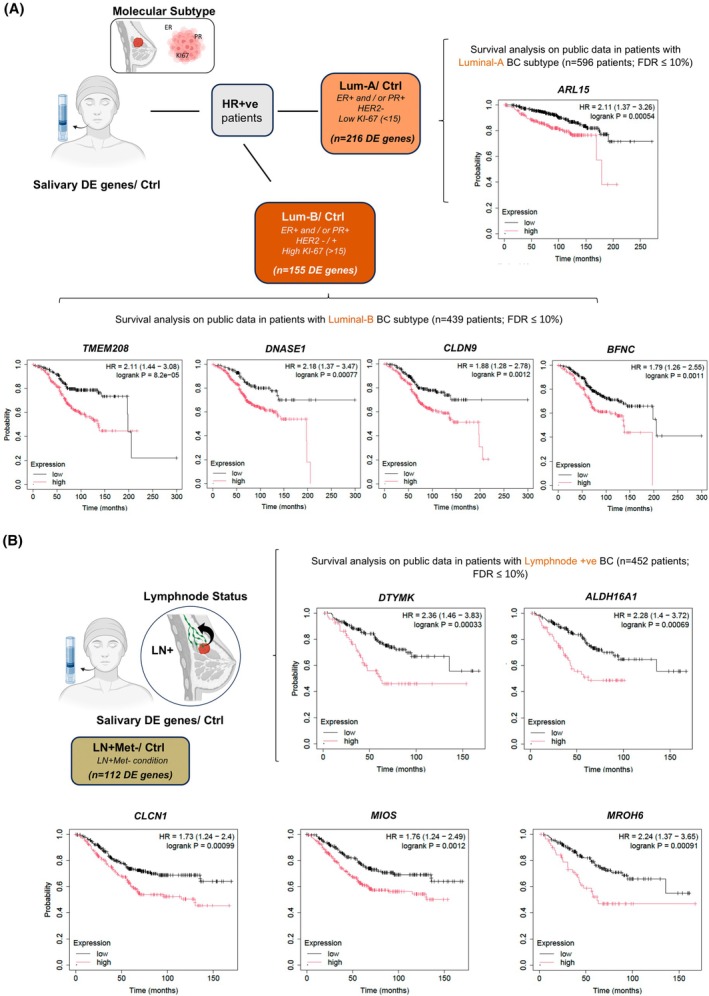
Prognostic significance of salivary upregulated genes in clinically relevant subtypes of breast cancer (BC). Kaplan–Meier survival analysis of the top prognostic salivary genes [FDR < 10%] identified among upregulated salivary gene profiles, evaluated for their association with overall survival in BC patients stratified by clinical subtypes. The analysis was performed using public datasets for patients with Luminal A (Lum‐A), Luminal B (Lum‐B) and lymph node‐positive (LN+) conditions, respectively. Kaplan–Meier plots: HR denotes hazard ratio; curves are stratified by expression (high = red, low = black) hazard ratio with 95% confidence interval (CI), and log‐rank *P* value are shown. (A) For salivary genes upregulated in Lum‐A (Lum‐A/Ctrl, *n* = 216), ARL15 was the most significant prognostic gene, with high expression correlating with reduced overall survival (hazard ratio = 2.11, 95% CI: 1.37–3.26, FDR < 10%) among Lum‐A patients. For Lum‐B upregulated salivary genes (Lum‐B/Ctrl, *n* = 155 salivary DE genes), *TMEM208, DNASE1, CLDN9,* and *BFNC* showed the strongest association with poor prognosis (hazard ratio range: 1.79–2.18, FDR < 10%) among Lum‐B patients. (B) Among salivary genes upregulated in the LN+ group (LN+Met‐/Ctrl; *n* = 112 salivary DE genes), high expression of *DTYMK,* *ALDH* *16A1*, *CLCN1*, *MIOS*, and *MROH6* was significantly associated with decreased survival (hazard ratio range: 1.73–2.36, FDR < 10%) as observed in patients with LN+ conditions.

Survival analysis of luminal‐associated salivary genes was next performed among HR+ patients using public data. In the Lum‐A subgroup (*n* = 596 patients), *ARL15* was identified as the only gene associated with poor prognosis among 216 salivary Lum‐A upregulated genes (hazard ratio = 2.11, FDR < 10%) (Fig. 5A). In contrast, analysis within the Lum‐B subgroup (*n* = 439 patients) revealed four genes among 155 salivary Lum‐B upregulated genes (e.g., *TMEM208*, *DNASE1*, *CLDN9*, and *BFNC*) that were linked to poor outcomes, with hazard ratios ranging from 1.79 to 2.18 (FDR < 10%) (Fig. 5A). These results are consistent with the generally more aggressive clinical behavior of Lum‐B compared with Lum‐A.

Among the salivary DE genes to our LN+Met‐ cohort compared with controls (*n* = 112, genes), five genes (*DTYMK*, *ALDH16A1*, *CLCN1*, *MIOS*, and *MROH6*) were found to be significantly associated with poor overall survival (Fig. 5B). Hazard ratios ranged from 1.73 to 2.36 (FDR < 10%) based on the analysis of a public cohort of 452 patients with LN+ status. Notably, three of these genes, *MROH6*, *DTYMK*, and *MIOS*, were also among the salivary LN+ gene signature identified in earlier analyses (Fig. 4E), further reinforcing their potential relevance and implication in the LN+ phenotype (Fig. 5B). The concordance between salivary RNA profiles and tumor survival trends in matched clinical contexts, such as molecular subtype or nodal status, strongly suggests that salivary transcriptomics can reflect key aspects of tumor biology.

### 3.7 Posttreatment‐related salivary gene expression changes in BC patients

**Fig. 6:**
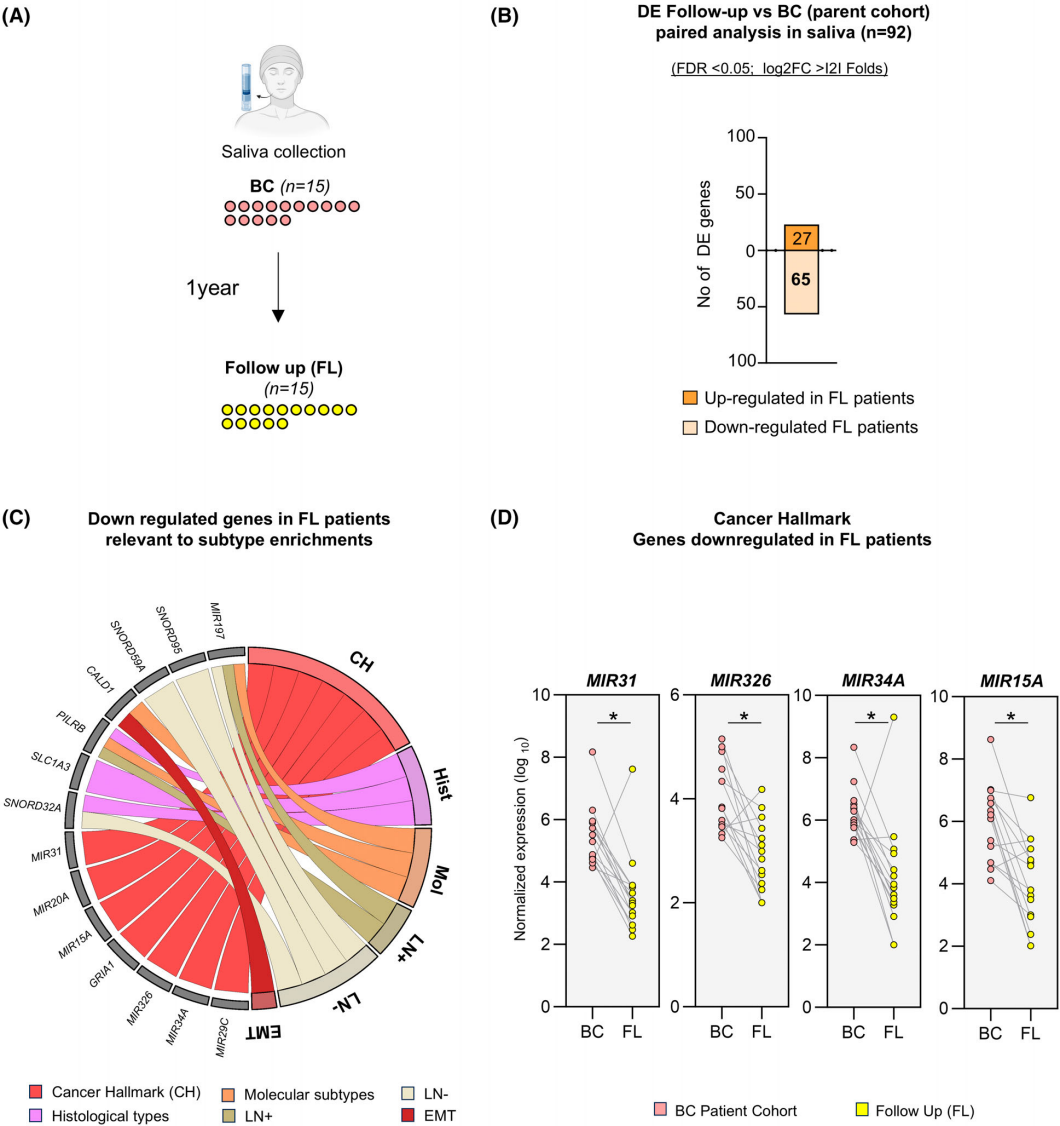
Differential gene expression analysis in saliva samples from breast cancer (BC) patients over a 1‐year follow‐up. (A) Schematic representation of the study design depicting saliva collection from BC patients (*n* = 15) at baseline and a paired follow‐up (FL) collection after 1 year (*n* = 15). (B) Paired differential expression (DE) analysis revealed changes in gene expression between the follow‐up cohort (*n* = 15) and the initial BC cohort (*n* = 15). The bar chart shows the number of significant DE genes [false discovery rate (FDR) < 0.05, log_2_ fold change (log_2_FC) > I2I], with 65 genes downregulated and 27 genes upregulated in follow‐up patients compared with the baseline BC cohort. (C) Chord diagram illustrating associations between downregulated genes in saliva samples from FL patients and specific cancer‐relevant categories, including Cancer Hallmarks (CH), molecular subtypes (Mol), histological types (Hist), lymph node positive (LN+), lymph node negative (LN‐) and epithelial–mesenchymal transition (EMT) upregulated in the parent cohort. (D) Dot plots display expression patterns of example cancer hallmark microRNAs downregulated in FL patients with (FDR < 0.05, log_2_FC > I2I). Each dot represents the gene's expression in an individual patient, with BC patient samples marked in light red and FL samples indicated in light yellow. Significance is denoted by * (FDR‐adjusted P < 0.05)

To investigate treatment‐related molecular changes, a follow‐up (FL) study was conducted involving 15 BC patients from the original cohort of 40 participants. Saliva samples were collected approximately 1 year after diagnosis, following primary tumor resection and adjuvant treatment with chemotherapy or radiotherapy aimed at reducing residual disease and recurrence risk (Fig. 6A). Paired differential gene expression analysis identified 92 DE genes between the baseline BC patients and FL cohorts (FDR < 0.05, log_2_FC > I2I). Among these genes, 27 were upregulated and 65 were downregulated in FL samples (Fig. 6B and Table S11). Notably, seven of the downregulated genes were cancer hallmark genes, predominantly comprising miRNAs, such as *MIR31*, *MIR326*, *MIR29C*, *MIR34A*, *MIR15A*, and *MIR20A*. Furthermore, *CALD1*, an EMT‐associated gene together with genes, such as *SNORD32A*, *MIR197*, *PILRB*, and *SLC1A3*, previously upregulated in histological or molecular subtypes, showed consistent downregulation in FL patient saliva posttreatment (Fig. 6C,D and Table S11). These changes hint at treatment‐driven molecular reprogramming distinct from baseline subtype transcriptional profiles. These findings highlight salivary transcriptomics as a promising noninvasive strategy for tracking treatment‐related molecular dynamics in BC, warranting validation in larger, independent cohorts to confirm its clinical utility.

### 3.8 Enrichment of novel genes in BC salivary transcriptome

**Fig. 7:**
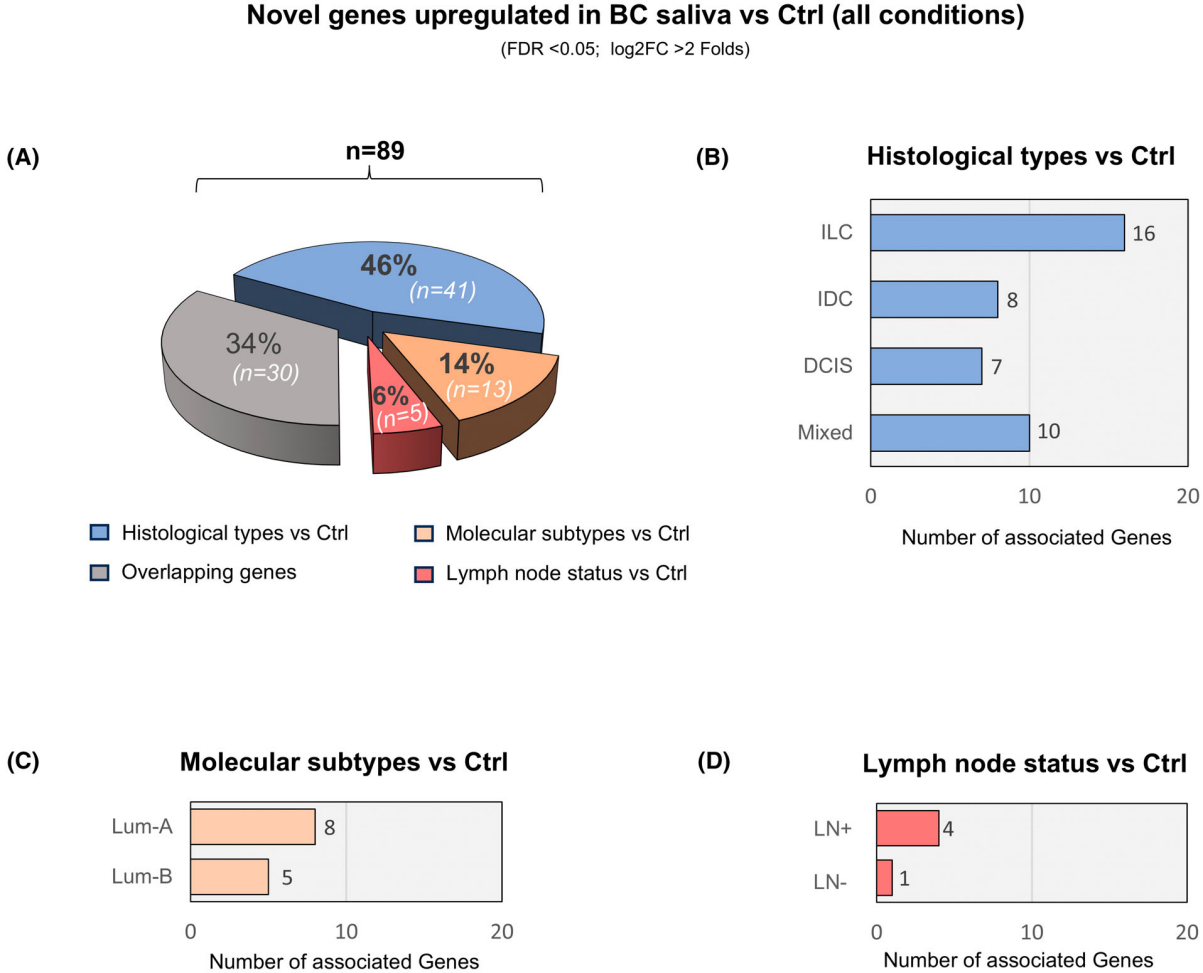
Enrichment and distribution of novel salivary genes across breast cancer (BC) subtypes. (A) A total of 89 novel Ensembl‐annotated genes (ENSG IDs) identified in saliva were differentially expressed (DE) BC cases versus controls. The pie chart shows the proportional distribution of 89 novel, significantly upregulated salivary genes, categorized by the clinical subgroup comparisons in which they were identified. Of these genes, 46% (*n* = 41) were associated with histological types, 14% (*n* = 13) with molecular subtypes, and 6% (*n* = 5) with lymph node (LN) status, while the remaining 34% (*n* = 30) were found to overlap between these comparison groups. (B–D) Bar charts detailing the distribution of the novel, significantly upregulated genes within specific clinical comparisons. The breakdown of 41 genes from the histological comparison shows the highest enrichment in invasive lobular carcinoma (ILC, *n* = 16), followed by mixed histologies (*n* = 10), invasive ductal carcinoma (IDC, *n* = 8), and ductal carcinoma *in situ* (DCIS, *n* = 7). Among molecular subtypes, a greater number of unique novel genes were enriched in Luminal A (Lum‐A) (*n* = 8) compared with Luminal B (Lum‐B) (*n* = 5). Analysis by LN status revealed that more novel genes were associated with LN+ patients (*n* = 4) than LN‐ patients (*n* = 1).

Upon investigating upregulated genes in BC across histological, molecular, and LN classifications compared with healthy controls (Figs 2A,B, 3A and 4A) we identified 89 novel (uncharacterized) salivary genes, annotated by unique Ensembl gene IDs (Fig. 7A and Table S12). Among these, 46% (*n* = 41) of the novel genes were exclusively associated with histological types (IDC, ILC, DCIS, and mixed vs. Ctrl), while 14% (*n* = 13) were identified in molecular subtype comparisons (HR+ subtypes Lum‐A and Lum‐B vs. Ctrl), and 6% (*n* = 5) were enriched based on LN status (LN+, LN‐ vs. Ctrl). Notably, 34% (*n* = 30) of the genes overlapped between these subgroups, reflecting shared upregulation patterns across clinical contexts. Further breakdown of histology‐related genes revealed that most genes were associated with ILC (*n* = 16), followed by mixed (*n* = 10), IDC (*n* = 8), and DCIS (*n* = 7) (Fig. 7B and Table S12). Among molecular subtypes, Lum‐A showed enrichment for eight unique genes, while Lum‐B was associated with five (Fig. 7C and Table S12). DE linked to LN status showed a predominance in LN+ patients (*n* = 4) compared with LN‐ (*n* = 1) (Fig. 7D and Table S12). These findings underscore the potential of salivary transcriptomics to capture novel, clinically relevant gene signatures across BC types, offering a promising noninvasive approach for biomarker discovery and patient stratification.

## 4 Discussion

Saliva has emerged as a promising noninvasive biofluid for BC diagnostics, offering advantages over other traditional screening methods, such as accessibility, low cost, noninvasiveness, and a rich molecular composition spanning transcripts, proteins, and metabolites [50, 51, 52]. While mammography remains the current gold standard, it presents some limitations in sensitivity, particularly in dense breast tissue, and its high false‐positive rates have driven the need for complementary diagnostic strategies [53, 54]. Recent developments, including AI‐assisted mammography, have significantly improved accuracy; however, accessibility remains an issue due to high initial costs and the requirement of specialized equipment and trained personnel. Tissue biopsy remains the reference for tumor diagnosis, but it is time‐consuming and lacks the convenience of noninvasive saliva sampling for routine monitoring. On the contrary, blood‐based liquid biopsies, though informative, involve invasive sampling and complex and costly processing, limiting their scalability in low‐resource settings [55, 56, 57]. Compared to these methods, saliva‐based RNA profiling offers a practical, patient‐friendly alternative, particularly suitable for repeated sampling and longitudinal monitoring. Our application of high‐throughput RNA sequencing provides a broader, unbiased transcriptomic view of RNAs relevant to BC diagnostics [58, 59]. In this study, we present a comprehensive salivary transcriptomic analysis in BC using high‐throughput RNA sequencing. By integrating diverse RNA species, including miRNAs, lncRNAs, and coding transcripts, and implementing stringent microbial filtering using dedicated bioinformatics tools, our approach enabled a streamlined, global snapshot of salivary gene expression in both healthy and BC‐afflicted individuals. Crucially, this work highlights the underutilized potential of saliva as a noninvasive diagnostic medium for BC detection, subtype classification, and therapeutic monitoring.

A critical advancement of this study lies in the identification and validation of robust RNA signatures that are both globally relevant and specific to histological types and molecular subtypes of BC. A robust 10‐gene salivary signature was found to be associated with BC presence and exhibited diagnostic relevance, with components, such as *ASF1A* and *MIR197* reflecting known roles in chromatin regulation and tumorigenesis [60, 61, 62]. While the roles of genes such as *PELO‐AS1* and *TPRN* are only starting to emerge in other cancers, their overexpression in the saliva of BC patients presents a strong rationale for future mechanistic exploration in this context [41]. The identification of stably expressed RNAs, including *MYT1L*, *MUC5AC*, and *NOP56*, further offers practical utility for normalization in future salivary assays.

In this study, we identified a 12‐gene salivary RNA signature comprising three transcripts each that robustly distinguish IDC, ILC, DCIS, and mixed (invasive/noninvasive) BC from healthy controls. Notably, this study identifies and validates salivary *MUC16* (encoding the cancer antigen CA125) as a IDC‐enriched biomarker with clinical relevance. Elevated salivary *MUC16* expression in IDC patients closely mirrors its upregulation in tumor tissue, nipple discharge, and serum reported in previous studies [63, 64, 65]. This finding is particularly compelling given that CA125 is already an established biomarker in clinical use for ovarian cancer, where it plays a central role in diagnosis, monitoring, and recurrence detection. Beyond ovarian malignancies, elevated CA125 levels have been documented in pancreatic, endometrial, and nonsmall cell lung cancers [66, 67, 68, 69], further substantiating its role as a broadly relevant tumor‐associated glycoprotein. Our demonstration of *MUC16* overexpression in IDC saliva, along with its validated upregulation in an independent cohort, supports salivary *MUC16* as a noninvasive, diagnostically relevant biomarker for IDC. Similarly, markers such as *HDAC3*, *ZAN*, and *PDE5A* were preferentially expressed in ILC patient saliva, underscoring the potential for salivary RNA to support histological subtype stratification. We also identified distinct 4‐gene salivary profiles for Luminal A and Luminal B subtypes. Lum‐A markers such as GRIA2, *ROGDI*, *PLCD4*, *MAPK9*, and *PCDH1* are linked to low‐grade, slow‐cycling tumors with strong epithelial cohesion and enhanced DNA repair [70, 71, 72, 73]. In contrast, Lum‐B markers *CNTNAP2,* *FOXM1*, *IGF2BP1*, *CLDN9*, and *LTBP1* are associated with rapid proliferation, EMT, and invasive phenotypes, reflecting the pro‐invasive microenvironment typical of Lum‐B subtype [74, 75, 76, 77, 78, 79, 80]. However, these findings should be interpreted with caution, as Luminal B breast cancers while generally characterized by higher proliferation can exhibit considerable heterogeneity in proliferative behavior, particularly in cases with high ER but low PR, which may indicate a less aggressive biological phenotype [81, 82]. These genes specific to IDC, ILC, and HR+ subtypes (Lum‐A and Lum‐B) demonstrated consistent enrichment in public tumor datasets of corresponding histological and molecular subtype. Importantly, these genes also showed significant enrichment in salivary samples from an independent validation cohort, further reinforcing their biological relevance and diagnostic potential.

Beyond subtype identification, our study highlights a distinct salivary gene expression signature associated with LN+ breast cancer. This 10‐gene panel, including known drivers of metastasis such as *CDCA3*, *DTYMK*, *KIF21A*, *SEPTIN9*, and *MPP7* [83, 84, 85, 86, 87], was selectively enriched in saliva from LN+ patients and showed consistent overexpression in primary tumor datasets from LN+ versus LN‐ cases. This reproducibility across independent sources reinforces the biological linkage of the signature to nodal involvement. Importantly, this LN+ signature appears to reflect early metastatic potential rather than long‐term prognosis, indicating its utility as a diagnostic biomarker for regional spread. Our exploratory survival analyses identified a separate set of salivary RNA transcripts with potential prognostic relevance. For example, elevated salivary expression of *ARL15* in Luminal A and *CLDN9* or *TMEM208* in Luminal B subtypes was associated with poorer overall survival, consistent with their roles in proliferation and epithelial barrier disruption. In LN+ patients, salivary expression of *DTYMK*, *MIOS*, and *MROH6*, distinct from the diagnostic LN+ signature, correlated with reduced survival, suggesting that selected genes may serve dual roles in metastatic identification and risk stratification, though further validation is needed. Additionally, we also observed dynamic transcriptomic shifts posttreatment, with a notable downregulation of cancer‐associated miRNAs (*MIR31*, *MIR15A*, and *MIR29C*) and other subtype‐specific genes in follow‐up samples. While it is difficult to draw definitive conclusions without prolonged longitudinal validation, the observed decrease in these genes, many of which have been linked to cancer progression, immune modulation, or therapy resistance, suggests a possible biological response to chemotherapy or radiotherapy [88, 89, 90, 91]. Although exploratory in nature and limited by sample size, these findings hint at the feasibility of salivary profiling as a tool to monitor therapeutic efficacy and remission.

Previous salivary biomarker studies have largely relied on targeted assays, such as qRT‐PCR or ELISA to detect a limited number of predefined molecules (e.g., *miR‐21*, CA15‐3, and HER2). While useful, these approaches inherently restrict the scope of discovery [18, 21, 22, 23]. In contrast, our study leverages an RNA‐seq‐based approach to enable comprehensive, unbiased transcriptomic profiling, allowing simultaneous identification of both known and novel salivary biomarkers without prior assumptions. Building on this capability, our analysis uncovered several previously uncharacterized salivary transcripts that are significantly upregulated across various conditions in BC patients. These novel genes, with currently unknown functions and no prior documented association with BC, may represent unrecognized elements of tumor biology. Their consistent salivary expression suggests potential relevance in the disease process and highlights new, unexplored avenues for biomarker discovery and functional investigation. A key strength of our study lies in its robust validation strategy. Despite a limited sample size, the consistent findings provide robust support for our results. Additionally, the use of unstimulated saliva in our study, a method endorsed by recent meta‐analyses for enhanced diagnostic accuracy [21], strengthens the methodology of our study. Importantly, our work addresses critical gaps identified in recent reviews, such as the absence of subtype‐stratified analyses and standardized transcriptomic methods [18]. We have made a concerted effort to address these gaps by focusing on histological types and HR+ subtypes of BC. However, due to the limited cohort size, we were unable to robustly analyze HR‐ subtypes. Despite this limitation, our subtype‐stratified approach still represents a meaningful step toward saliva‐based biomarker discovery.

To strengthen the clinical relevance of our findings, we further compared salivary transcriptomic profiles to established BC diagnostic signatures and tumor‐derived circulating markers. Comparison with 33 established multigene signatures used in BC diagnostic panels revealed notable concordance, with 8% (i.e. 244 of 3048) of consistently expressed salivary genes in our BC cohort (Table S4) overlapping with 1895 genes from these panels (Fig. S6 and Table S13). This includes canonical markers like *MKI67*, *ESR1*, and *PGR* integral to existing diagnostic assays, such as Oncotype DX and MammaPrint, while not differentially expressed in our dataset, were nonetheless reliably detected in saliva. This indicates that saliva harbors stable, informative transcripts routinely used in clinical decision‐making. Furthermore, 117 out of 892 highly expressed salivary DE genes in our study also show overexpression (> 1 FC) in BC blood circulating tumor cells (CTCs). This is a meaningful overlap (13.1%) particularly between biofluids with distinct collection methods and biomolecular content (Table S14). It reinforces the potential of salivary RNA to serve as a viable indicator of tumor biology and a clinically useful biospecimen in BC research and diagnostics.

Saliva‐based RNA diagnostics offer a unique combination of affordability, scalability, and ease of use. Unlike imaging, biopsies, or blood‐based assays that require significant infrastructure and specialized personnel, saliva‐based kits could be developed at low cost, allowing generalized screening, especially in low‐income regions where access to standard BC diagnostics is limited, which would improve BC management. Additionally, the growing public familiarity with saliva‐based health testing may help overcome acceptance barriers and streamline implementation at the population level [92, 93]. Despite these strengths, the study acknowledges key limitations, including its modest sample size and unicentric design. Broader validation across multi‐ethnic, multicentered cohorts is essential to ensure generalizability and clinical reliability. Notably, the mean age differences between BC and control groups, although consistent with the epidemiology of HR+ breast cancer, may introduce age‐related transcriptional variance, particularly in small cohorts. Going forward, additional strong validations in large cohorts of the above‐identified signatures may position saliva‐based diagnostics as a versatile tool for BC screening, subtyping, metastasis, risk stratification, and posttreatment monitoring. Overall, this approach could significantly improve access to personalized cancer care in under‐resourced settings.

## 5 Conclusion

This study offers a proof‐of‐concept for the application of salivary RNA sequencing as a noninvasive and biologically informative platform for BC biomarker discovery. By integrating whole‐transcriptome profiling with validation across clinical subtypes and tumor‐relevant datasets, we provide preliminary evidence that saliva harbors diagnostically and potentially prognostically relevant transcripts reflective of BC biology. The ability to noninvasively capture gene expression signatures associated with histology, HR status, lymph node involvement, and therapeutic response positions saliva as a compelling candidate for scalable diagnostics, particularly in settings where access to conventional tools is limited. We acknowledge that larger, multi‐institutional studies with diverse populations will be essential to validate and refine these candidate biomarkers. Nonetheless, this work establishes a foundational framework for the development of saliva‐based assays that could broaden access to precision oncology and support longitudinal disease monitoring in a cost‐effective, patient‐friendly manner.

## Conflict of interest

The authors declare no conflict of interest.

## Author contributions

MAB, NR, and WT contributed to the conceptualization. LD contributed to the collection and assembly of the samples. MAB, MS, WT, and IP contributed to the ethical file preparation. WT and LD contributed to the clinical study support. AJ, MS, NR, and MAB contributed to the RNA isolation optimization and validation analysis. IP, NR, LD, and WT contributed to the patient data collection and curation. NR and MAB contributed to the supervision. EE and NR contributed to the bioinformatic analysis. NR, EE, IP, MAB, RQ, and KT contributed to the data interpretation and discussion. NR and EE contributed to the visualization. NR contributed to the writing—original draft. NR, MAB, IP, RQ, and KT contributed to the writing—review and editing. All authors reviewed the results and approved the final version of the manuscript.

## Supporting information


**Table S1.** Clinical and demographic data of the study participants. This table provides a detailed description of the patient cohorts, including disease characteristics of breast cancer (BC) patients (n = 40), follow‐up patients (n = 15), and an independent validation cohort of BC (n = 20). Information about control individuals is also included.

**Table S2.** Bacterial genome identifiers used for filtering. This table lists the identifiers of bacterial genomes employed in the study to filter the dataset.

**Table S3.** List of primer sequences used for experimental validation of salivary genes examined in this study.

**Table S4.** List of genes consistently expressed in saliva. This table lists all 3048 genes expressed across all samples (BC and controls) along with their normalized expression values.

**Table S5.** Top stable genes in saliva. This table presents the 50 most stable salivary genes, along with their associated stability metrics, including the M value, coefficient of variation (CV), and combined rank, for genes stable between BC and control groups and those stable only in controls.

**Table S6.** Differentially expressed (DE) genes between BC and control groups. This table provides the list of DE genes identified between BC patients and controls, with false discovery rate (FDR) < 0.05, including their log2 fold change (log2FC) and adjusted p‐values (FDR). Genes with log2FC >2 and false discovery rate (FDR) < 0.05 are highlighted in gray.

**Table S7.** Weighted gene co‐expression network analysis (WGCNA) results. The table details module information, including module color and related data on the genes associated with each module.

**Table S8.** DE genes (FDR <0.05) from saliva samples of patients with invasive ductal carcinoma (IDC), invasive lobular carcinoma (ILC), ductal carcinoma *in situ* (DCIS), and mixed histologies, compared to healthy controls. Each comparison includes log2 fold change (log2FC) and adjusted p‐values (FDR). Genes with log2FC >2 and false discovery rate (FDR) < 0.05 are highlighted in gray. Additionally, list of 202 associated cancer hallmark genes along with their distribution across hallmark pathways has been included.

**Table S9.** DE genes (FDR <0.05) from saliva samples of patients with Luminal A and Luminal B breast cancer compared to healthy controls. Each comparison includes log2 fold change and adjusted p‐values (FDR). Each comparison includes log2 fold change (log2FC) and adjusted p‐values (FDR). Genes with log2FC >2 and false discovery rate (FDR) < 0.05 are highlighted in gray. Additionally, list of 100 associated cancer hallmark genes along with their distribution across hallmark pathways has been included.

**Table S10.** DE genes (FDR <0.05) from saliva samples of breast cancer patients stratified by lymph node (LN) status. Comparisons include LN‐positive (LN+Met−) and LN‐negative (LN−Met−) patients versus healthy controls. Each comparison includes log2 fold changes (log2FC) and adjusted p‐values (FDR). Genes with log2FC >2 and false discovery rate (FDR) < 0.05 are highlighted in gray. Additionally, a list of 98 DE genes specific to LN+ condition is provided.

**Table S11.** DE genes (FDR <0.05) between baseline breast cancer patients and the same patients after 1 year of follow‐up (FL). The table includes both upregulated and downregulated transcripts, along with log2 fold changes (log2FC) and adjusted p‐values (FDR). Genes with log2FC >2 and false discovery rate (FDR) < 0.05 are highlighted in gray. A list of 7 cancer hallmark genes downregulated at follow‐up is included, along with their association to hallmark pathways.

**Table S12.** Novel salivary transcripts identified through RNA sequencing that lack prior functional annotation with their Ensembl gene IDs, genomic coordinates, transcript biotype, and any available NCBI descriptions. Genes with log2FC >2 and false discovery rate (FDR) < 0.05 are highlighted in gray. The distribution of these transcripts across DE lists pertaining to histological types, molecular subtypes, and LN status is also highlighted.

**Table S13.** Overlap of salivary genes with known breast cancer gene signatures. This table presents the overlap of salivary genes with 33 known BC multigene panels, showing the number of overlapping genes for each panel out of the 3048 salivary genes expressed in all 40 BC patient samples.

**Table S14.** List of salivary DE genes that also show >1‐fold enrichment in blood‐derived CTC datasets from breast cancer patients.

**Fig. S1.** Patient characteristic profiles across molecular subtypes of breast cancer.
**Fig. S2.** Exploration and validation of salivary breast cancer signatures.
**Fig. S3.** Epithelial–mesenchymal transition and upregulated salivary genes relevant to subtype‐specific patterns in Luminal A and Luminal B breast cancer.
**Fig. S4.** Lymph node‐positive and lymph node‐negative salivary signatures in tumor samples.
**Fig. S5.** Prognostic value of saliva‐derived DE genes in BC patients.
**Fig. S6.** Overlap of salivary RNA species with established breast cancer multigene signatures.

## Data Availability

The raw FASTA files for gene expression data generated and analyzed during this study have been deposited in the Array Express database with accession number: E‐MTAB‐14645.
