# Supplementary material for: Characterizing the salivary RNA landscape to identify potential diagnostic, prognostic, and follow‐up biomarkers for breast cancer
Source: Mol Oncol. 2025 Sep 1;20(2):282–306. doi: 10.1002/1878-0261.70101 (PMC12936433; doi:10.1002/1878-0261.70101)
Supplement: Supplementary file 15 — Fig. S1. Patient characteristic profiles across molecular subtypes of breast cancer. Fig. S2. Exploration and validation of salivary breast cancer signatures. Fig. S3. Epithelial–mesenchymal transition and upregulated salivary genes relevant to subtype‐specific patterns in Luminal A and Luminal B breast cancer. Fig. S4. Lymph node‐positive and lymph node‐negative salivary signatures in tumor samples. Fig. S5. Prognostic value of saliva‐derived DE genes in BC patients. Fig. S6. Overlap of salivary RNA species with established breast cancer multigene signatures. [file MOL2-20-282-s002.pdf]

Supplementary Figures (S1-S6)

Characterizing the salivary RNA landscape to identify potential diagnostic, prognostic and follow-up biomarkers for breast cancer.

Rajan Nicholas[1,*], Primac Irina[1], Etlioglu Emre[1], Debruyne Laurens [2], Janssen Ann[1], Sallam Magy[1], Tabury Kevin[1], Quintens Roel[1], Tjalma Wiebren [2], Benotmane Mohammed Abderrafi[1,*]

.

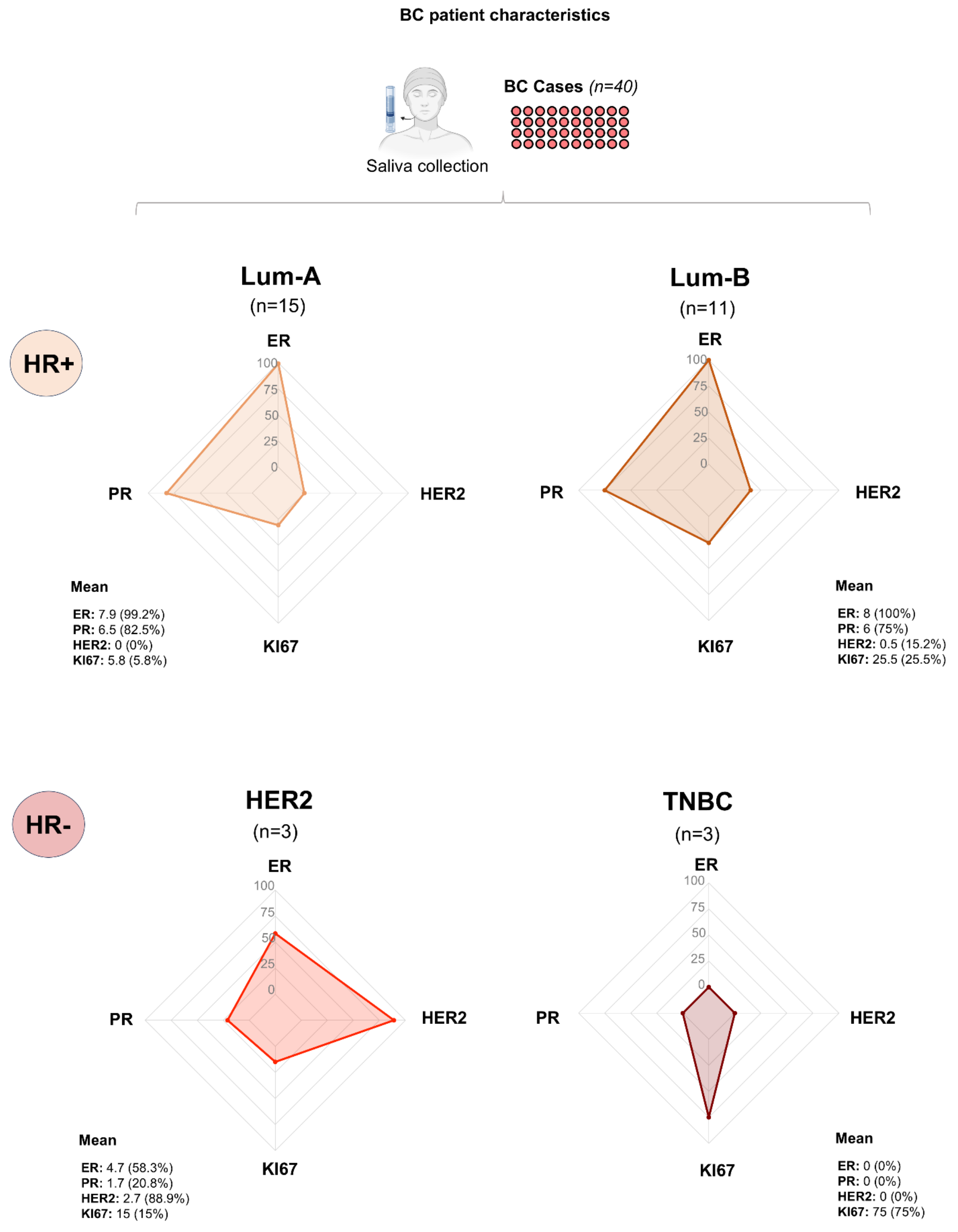

Supplementary Fig. S1: Patient characteristic profiles across molecular subtypes of breast cancer. Demographic and clinical characteristics of patients were stratified according to molecular subtypes of breast cancer (BC), including Luminal A (Lum-A), Luminal B (Lum-B), human epidermal growth factor receptor 2 (HER2)-enriched, and triple-negative breast cancer (TNBC). Subtypes were defined based on the expression status of estrogen receptor (ER), progesterone receptor (PR), human epidermal growth factor receptor 2 (HER2), and the proliferation marker Ki-67, in accordance with current clinical guidelines. Radar plots display the relative expression levels (normalized to 100%) of these key biomarkers in salivary samples derived from patients with distinct breast cancer subtypes. Tumors were grouped based on hormone receptor (HR) status into HR-positive (HR+, top row) and HR-negative (HR-, bottom row) categories. Within the HR+ group, Luminal A tumors (**top left**) were characterized by high ER and PR expression, HER2 negativity, and a low Ki-67 index, while Luminal B tumors (**top right**) exhibited ER positivity, high or variable PR expression, HER2 negativity or positivity, and a moderately elevated Ki-67 index (>15%), indicating a more proliferative phenotype. In the HR- group, HER2-enriched tumors (**bottom left**) showed strong HER2 expression with low to moderate ER and PR expression, along with a mean Ki-67 level of 15%. Conversely, TNBC tumors (**bottom right**) were defined by the absence of ER, PR, and HER2 expression and exhibited a high Ki-67 index, reflecting a highly proliferative and aggressive tumor biology.

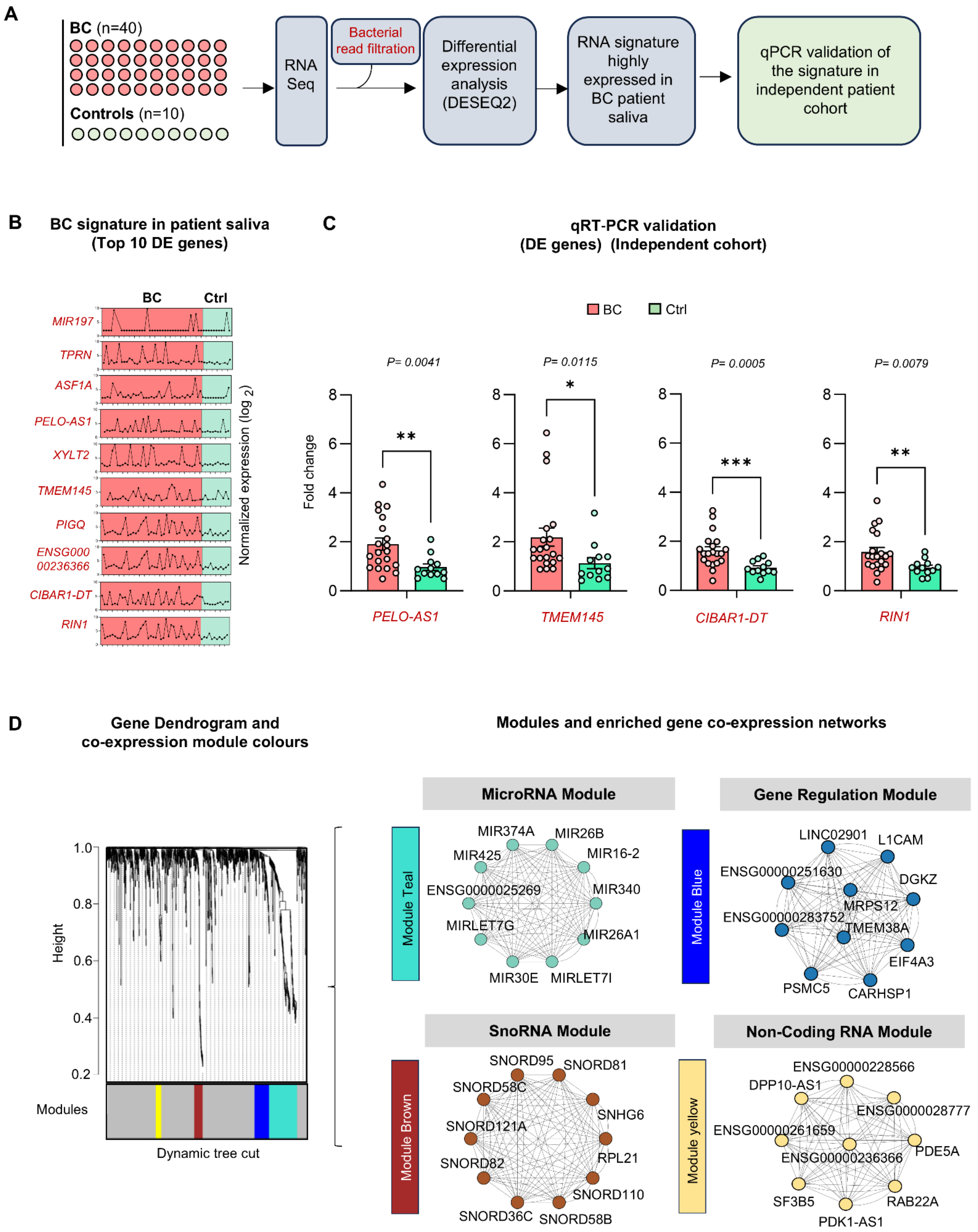
A
BC (n=40)
Controls (n=10)
RNA Seq
Bacterial read filtration
Differential expression analysis (DESEQ2)
RNA signature highly expressed in BC patient saliva
qPCR validation of the signature in independent patient cohort
B
BC signature in patient saliva (Top 10 DE genes)
BC
Ctrl
MIR197
TPRN
ASF1A
PELO-AS1
XYLT2
TMEM145
PIGQ
ENSG000 00236366
CIBAR1-DT
RIN1
Normalized expression (log 2)
C
qRT-PCR validation (DE genes) (Independent cohort)
BC
Ctrl
P= 0.0041
P= 0.0115
P= 0.0005
P= 0.0079
Fold change
**
*
***
**
PELO-AS1
TMEM145
CIBAR1-DT
RIN1
D
Gene Dendrogram and co-expression module colours
Height
Modules
Dynamic tree cut
Modules and enriched gene co-expression networks
MicroRNA Module
Module Teal
MIR374A
MIR26B
MIR425
MIR16-2
ENSG0000025269
MIR340
MIRLET7G
MIR26A1
MIR30E
MIRLET7I
Gene Regulation Module
Module Blue
LINC02901
L1CAM
ENSG00000251630
DGKZ
MRPS12
ENSG00000283752
TMEM38A
EIF4A3
PSMC5
CARHSP1
SnoRNA Module
Module Brown
SNORD95
SNORD81
SNORD58C
SNHG6
SNORD121A
RPL21
SNORD82
SNORD110
SNORD36C
SNORD58B
Non-Coding RNA Module
Module yellow
ENSG00000228566
DPP10-AS1
ENSG0000028777
ENSG00000261659
PDE5A
ENSG00000236366
SF3B5
RAB22A
PDK1-AS1

E

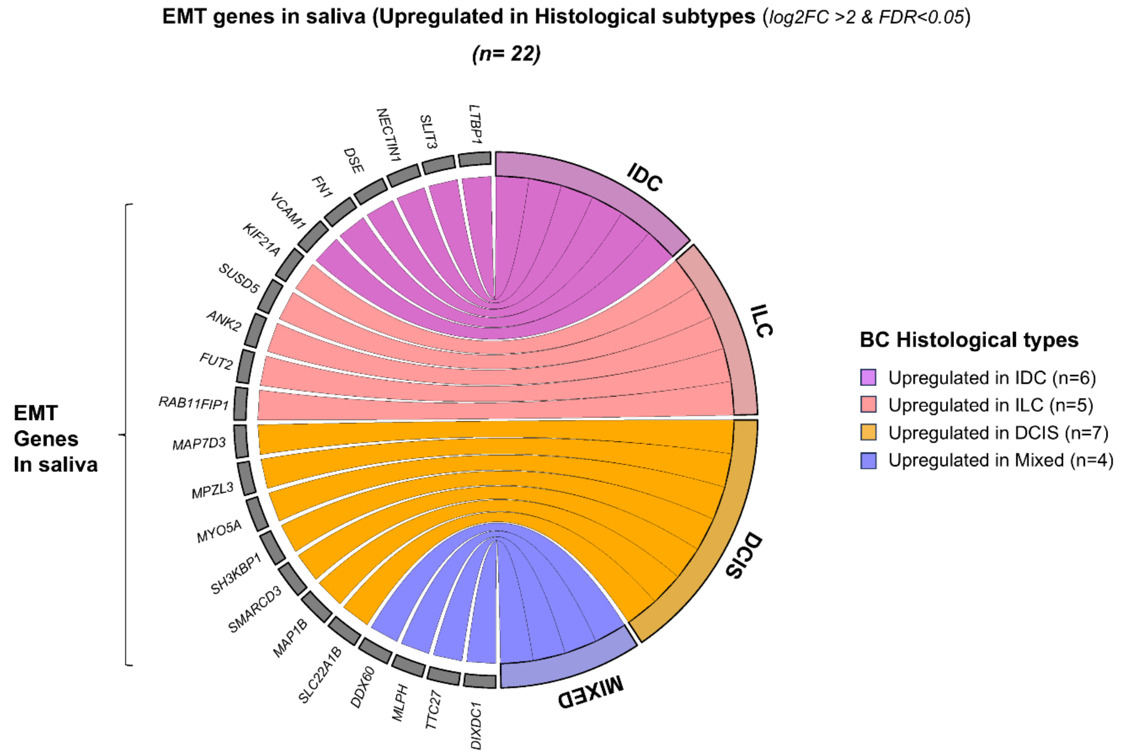
EMT genes in saliva (Upregulated in Histological subtypes (log2FC >2 & FDR<0.05)
(n= 22)
EMT Genes In saliva
LTBP1
SLIT3
NECTIN1
DSE
FN1
VCAM1
KIF21A
SUSD5
ANK2
FUT2
RAB11FIP1
MAP7D3
MPZL3
MYO5A
SH3KBP1
SMARCD3
MAP1B
SLC22A1B
DDX60
MLPH
TTC27
DIXDC1
IDC
ILC
DCIS
MIXED
BC Histological types
Upregulated in IDC (n=6)
Upregulated in ILC (n=5)
Upregulated in DCIS (n=7)
Upregulated in Mixed (n=4)

F

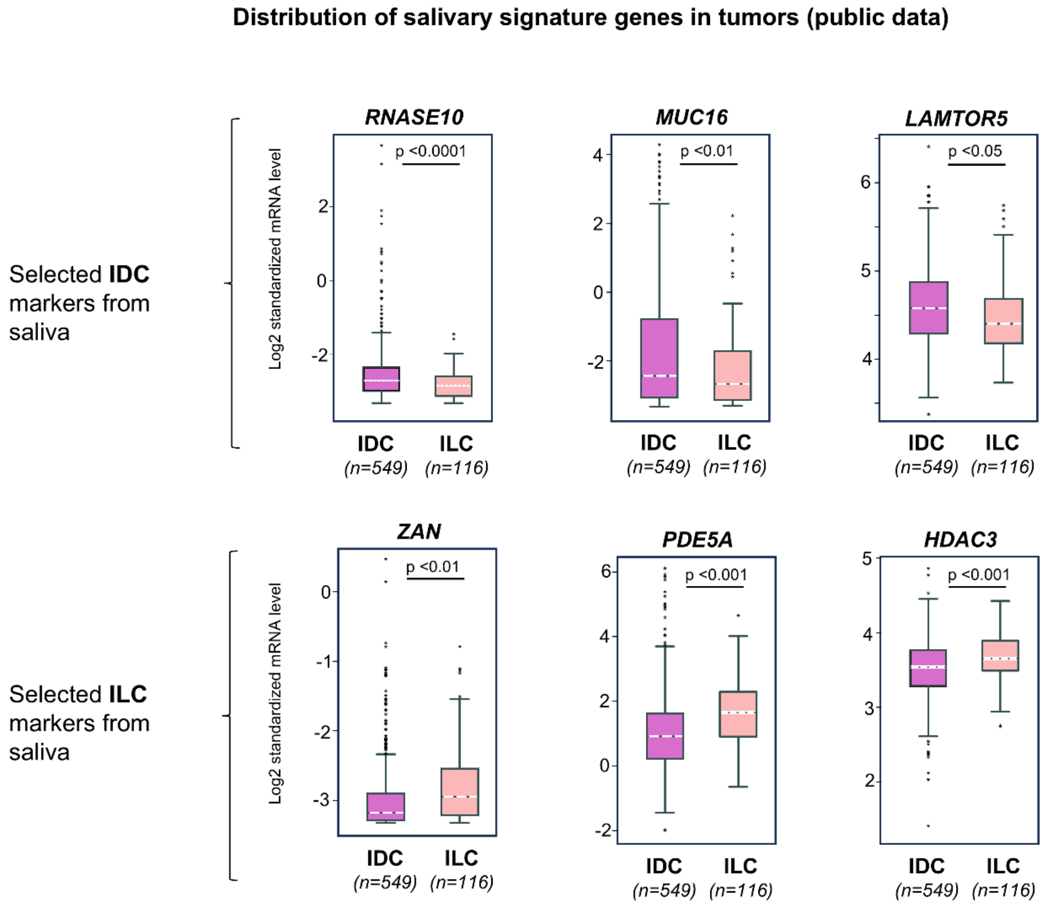
Distribution of salivary signature genes in tumors (public data)
Selected IDC markers from saliva
Selected ILC markers from saliva
Log2 standardized mRNA level
RNASE10
p <0.0001
MUC16
p <0.01
LAMTOR5
p <0.05
ZAN
p <0.01
PDE5A
p <0.001
HDAC3
p <0.001
IDC
(n=549)
ILC
(n=116)

Supplementary Fig S2: Exploration and validation of salivary breast cancer signatures **(A)** Schematic overview of the experimental design. RNA sequencing (RNA-seq) was performed, followed by differential expression analysis using DESeq2. RNA signatures highly expressed in breast cancer (BC) patient saliva were identified and subsequently validated via quantitative reverse transcription polymerase chain reaction (qRT-PCR) in an independent patient cohort. **(B)** Line expression plots of the top 10 upregulated genes in BC demonstrating distinct expression profiles between BC and control samples. Points connected by lines represent normalized expression values (log 10) for individual samples, with fluctuations indicating variability within each group (Red-BC and Green-Ctrl). **(C)** Validation of upregulated salivary genes in BC in independent cohort. Fold change denotes RNA expression relative to Ctrl, plotted as log2(fold change). Error bars indicate standard error of the mean (SEM). Statistical significance was assessed using a two-tailed Mann-Whitney U test, with p-values of 0.0041 for *PELO-AS1*, 0.0115 for *TMEM145,* 0.0005 for *CIBAR1-DT* and 0.0079 for *RIN1*. Significance is denoted by * (P < 0.05), ** (P < 0.005) and *** (P < 0.0005); **(D)** Gene dendrogram with four modules: (1) Teal 'MicroRNA' module; (2) Brown 'SnoRNA' module; (3) Blue 'Gene Regulation' module; (4) Yellow 'Non-Coding RNA' module. Modules reflect distinct regulatory RNA signatures relevant to BC biology. **(E)** Chord diagram showing the association between epithelial–mesenchymal transition (EMT)-related genes in saliva and histological types of BC, including invasive ductal carcinoma (IDC), invasive lobular carcinoma (ILC), ductal carcinoma in situ (DCIS), and mixed conditions. Each ribbon links individual genes to their predominant tumor subtype based on enrichment analysis. Color codes indicate IDC (purple), ILC (pink), DCIS (orange), and mixed (blue) subtypes. **(F)** Box plots display the tumor expression levels of 6 selected salivary upregulated genes associated with IDC (top) and ILC (bottom), as identified in Fig. 2F, using publicly available tumor datasets from IDC and ILC patients. Expression data were obtained from the Breast Cancer Gene-Expression Miner v5.2 (bc-GenExMiner v5.2), from IDC (n = 549) and ILC (n = 116) tumor samples. Global differences in gene expression were assessed using Welch's test, followed by Dunnett-Tukey-Kramer's post hoc test for pairwise comparisons. Adjusted p-values for statistically significant differences are indicated above the box plots. Box plots represent interquartile ranges (IQR), with the horizontal line indicating the median. Error bars indicate standard deviation (SD).

A

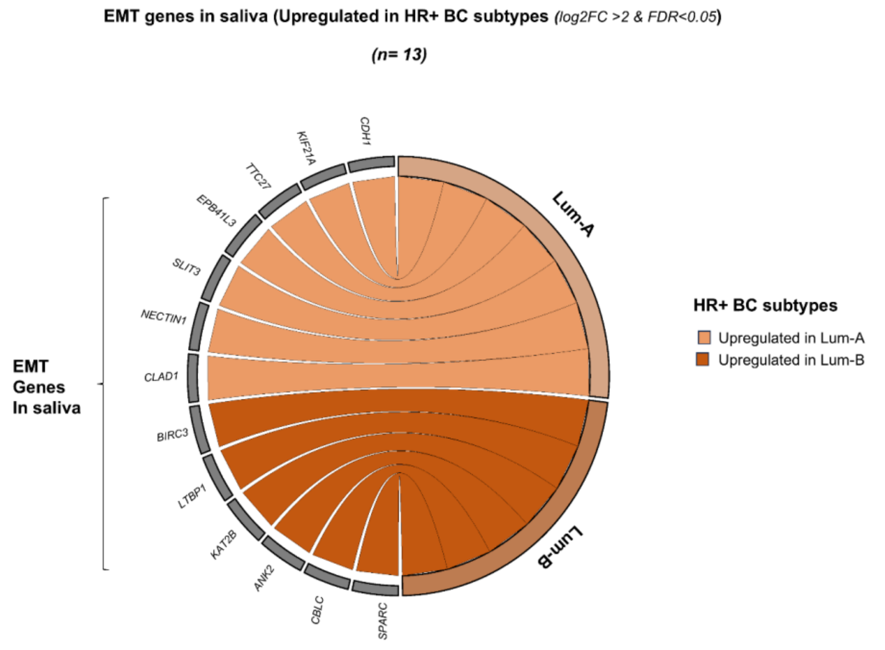

B

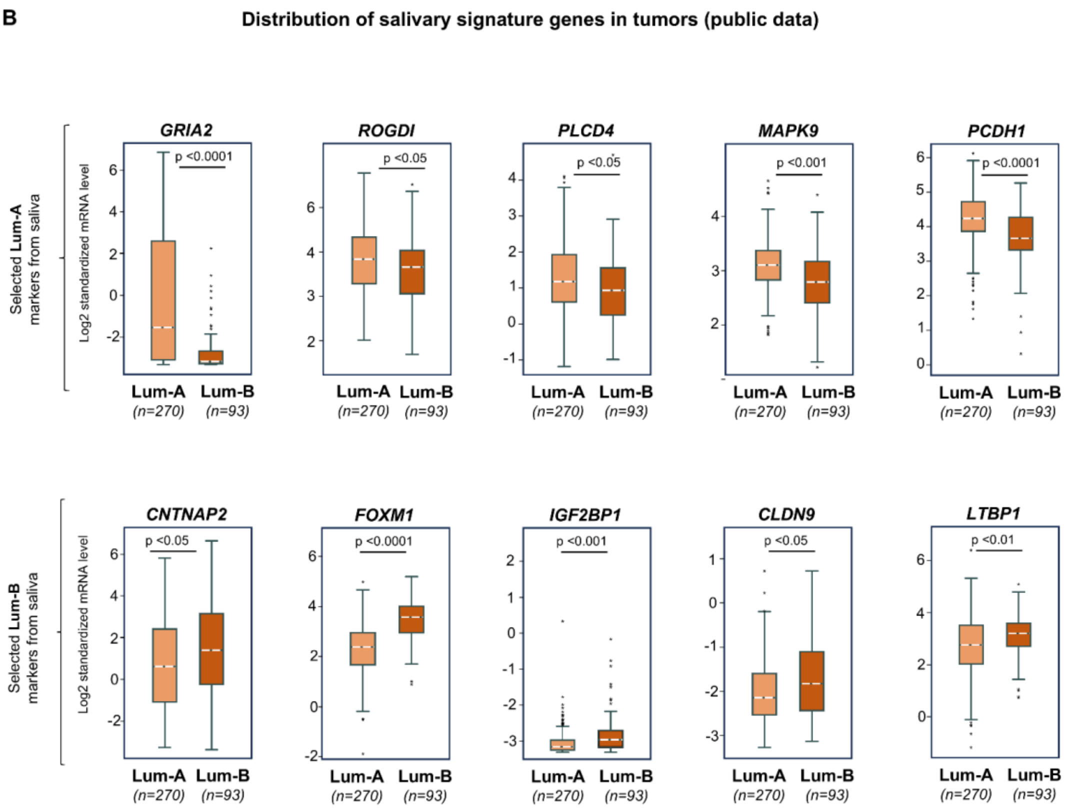

Supplementary Fig S3: Epithelial–mesenchymal transition and upregulated salivary genes relevant to subtype-specific patterns in Luminal A and Luminal B breast cancer. (A) Chord diagram illustrating the association between epithelial–mesenchymal transition (EMT)-related genes in saliva and molecular subtypes of breast cancer (BC), including Luminal A (Lum-A) and Luminal B (Lum-B) **(B)** Box plots display the tumor expression levels of 10 selected upregulated salivary genes associated with Lum-A (top) and Lum-B (bottom), (as identified in Fig. 3D), using publicly available tumor datasets from Lum-A and Lum-B patients. Expression data were obtained from the Breast Cancer Gene-Expression Miner v5.2 (bc-GenExMiner v5.2), from Lum-A (n = 270) and Lum-B (n = 93) tumor samples. Global differences in gene expression were assessed using Welch's test, followed by Dunnett-Tukey-Kramer's post hoc test for pairwise comparisons. Adjusted p-values for statistically significant differences are indicated above the box plots. Box plots represent interquartile ranges (IQR), with the horizontal line indicating the median. Error bars indicate standard deviation (SD).

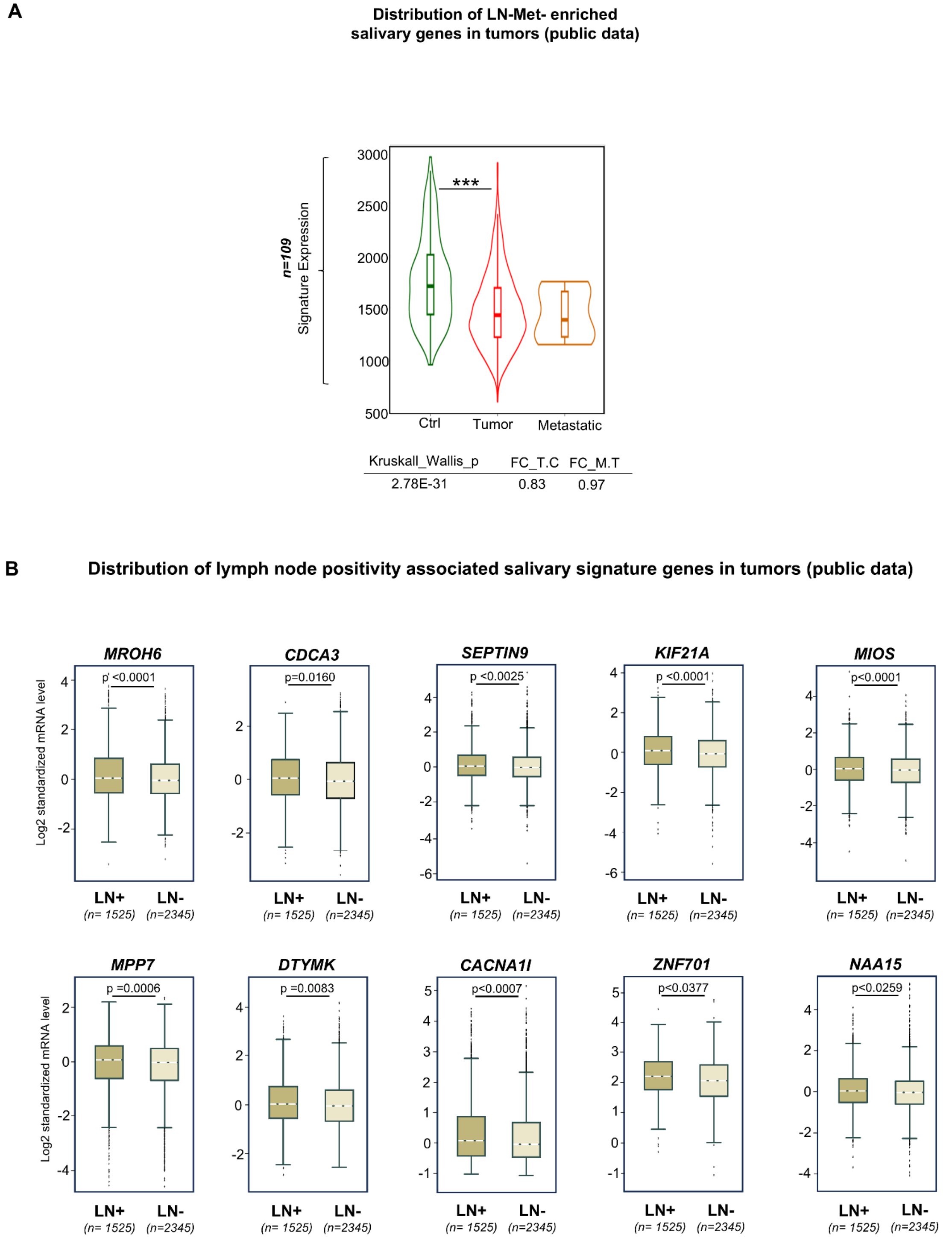

Supplementary Fig S4: Lymph node-positive and lymph node-negative salivary signatures in tumor samples (A) Violin plot comparing salivary genes enriched in lymph node-negative (LN-) and metastasis negative(Met-) (LN-Met-), in tumors using public tumor expression data. The Kruskal-Wallis test indicates a significant difference (p = 2.78E-31), with log2 fold changes of 0.83 (FC_T.C, tumor vs. control) and 0.97 (FC_M.T, metastatic vs. tumor), showing decreased expression of these genes in tumors and metastatic cases compared to controls (Ctrl). Significance is denoted by *** (P < 0.0005); **(B)** Box plots display the tumor expression levels of 10 selected salivary genes upregulated in lymph node-positive (LN+) condition (top and bottom), (as identified in Fig. 4E), using publicly available tumor datasets from patients with LN+ and LN- condition. Expression data were obtained from the Breast Cancer Gene-Expression Miner v5.2 (bc-GenExMiner v5.2), from LN+ (n = 1525) and LN- (n = 2345) tumor samples. Selected salivary genes enriched in the LN+ group relative to controls such as *MROH6, CDCA3, SEPTIN9, KIF21A, MIOS, MMP9, DTYMK, CACNA1l, ZNF701* and *NAA15* were also chosen based on their significantly higher expression in LN+ tumor samples compared to LN- samples (B, top and bottom). Global differences in gene expression were assessed using Welch's test, followed by Dunnett-Tukey-Kramer's post hoc test for pairwise comparisons. Adjusted p-values for statistically significant differences are indicated above the box plots. Box plots represent interquartile ranges, with the horizontal line indicating the median. Error bars indicate standard deviation (SD).

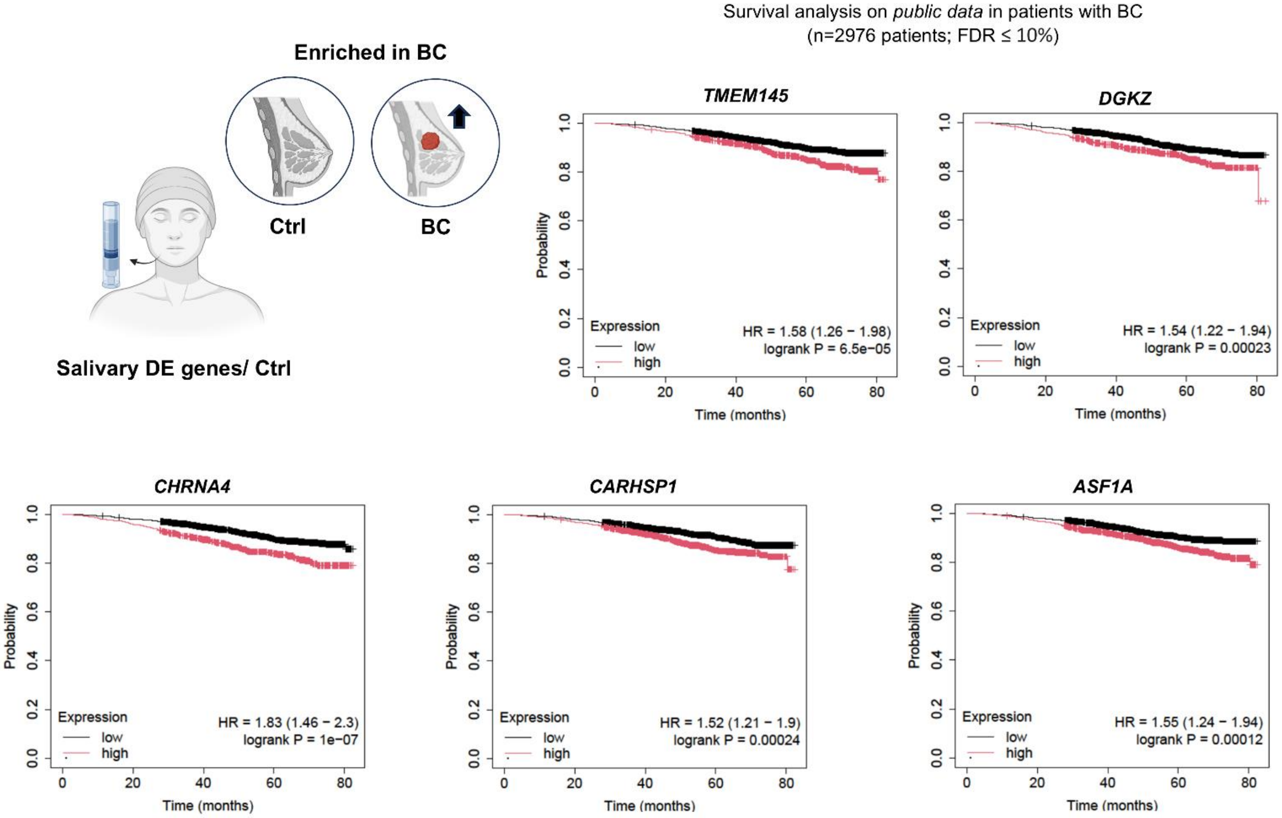

Supplementary Fig. S5: Prognostic value of saliva-derived DE genes in BC patients. Kaplan–Meier survival analyses of key genes identified as significantly upregulated in saliva of breast cancer (BC) patients compared to healthy individuals (Ctrl) and associated with poor prognosis (FDR ≤10%) in tumor transcriptomic datasets. The top five prognostic genes *TMEM145, DGKZ,* **(Top)** *CHRNA4, CARHSP1, and ASF1A* **(Bottom)** were evaluated for their association with overall survival across clinically relevant BC subtypes. Survival curves are stratified by gene expression levels (high: red; low: black), with hazard ratios (HR), 95% confidence intervals (CI), and log-rank P values shown.

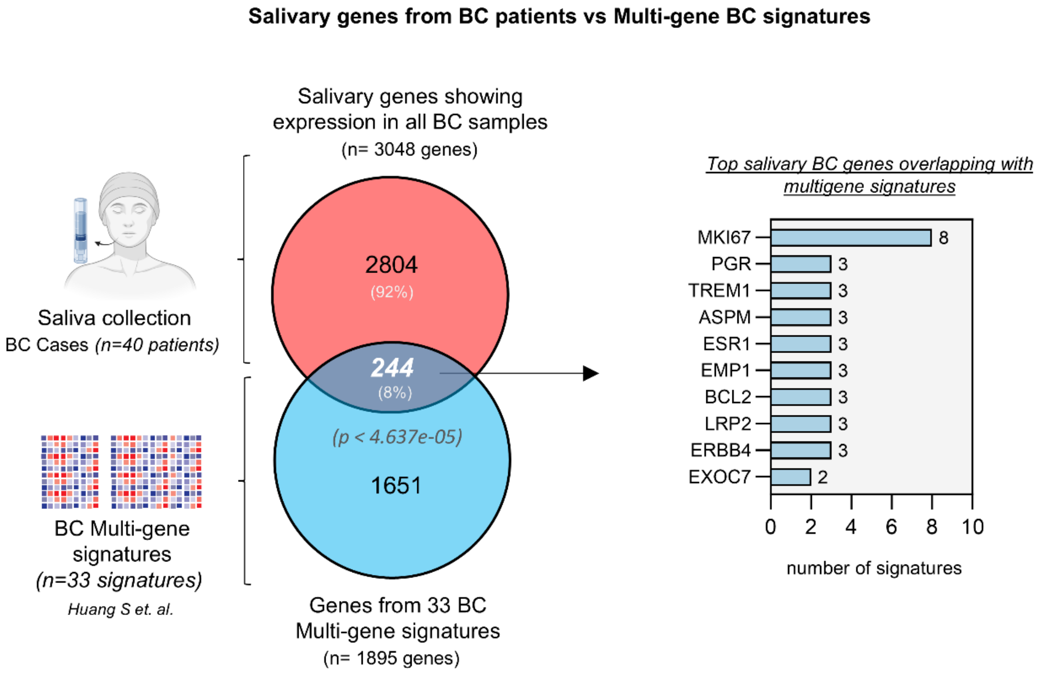

Supplementary Fig S6. Overlap of salivary RNA species with established breast cancer multigene signatures. Left: Venn diagram illustrating the overlap between 3,048 salivary genes with detectable expression (at least one normalized value) across all breast cancer (BC) samples and 1,895 genes derived from known BC multigene signatures. **Right:** Bar chart depicting the top genes from the salivary RNA profile that overlap with multiple BC gene signatures.
